# UCHL1 contributes to insensitivity to endocrine therapy in triple-negative breast cancer by deubiquitinating and stabilizing KLF5

**DOI:** 10.1186/s13058-024-01800-1

**Published:** 2024-03-11

**Authors:** Juan Li, Yu Liang, Shijie Zhou, Jie Chen, Chihua Wu

**Affiliations:** 1 Department of Breast Surgery, School of Medicine, Sichuan Provincial People’s Hospital, University of Electronic Science and Technology of China, Chengdu, China; 2 Department of Health Management & Institute of Health Management, School of Medicine, Sichuan Provincial People’s Hospital, University of Electronic Science and Technology of China, Chengdu, China; 3 State Key Laboratory of Biotherapy and Cancer Center, West China Hospital, Sichuan University, Chengdu, China

**Keywords:** UCHL1, KLF5, EGFR, TET1, TET3, ERα, Triple negative breast cancer

## Abstract

**Background:**

Ubiquitin carboxyl-terminal hydrolase L1 (UCHL1) is a deubiquitinating enzyme that regulates ERα expression in triple-negative cancer (TNBC). This study aimed to explore the deubiquitination substrates of UCHL1 related to endocrine therapeutic responses and the mechanisms of *UCHL1* dysregulation in TNBC.

**Methods:**

Bioinformatics analysis was conducted using online open databases. TNBC representative MDA-MB-468 and SUM149 cells were used for in vitro and *in-vivo* studies. Co-immunoprecipitation was used to explore the interaction between UCHL1 and KLF5 and UCHL1-mediated KIF5 deubiquitination. CCK-8, colony formation and animal studies were performed to assess endocrine therapy responses. The regulatory effect of TET1/3 on *UCHL1* promoter methylation and transcription was performed by Bisulfite sequencing PCR and ChIP-qPCR.

**Results:**

UCHL1 interacts with KLF5 and stabilizes KLF5 by reducing its polyubiquitination and proteasomal degradation. The UCHL1-KLF5 axis collaboratively upregulates *EGFR* expression while downregulating *ESR1* expression at both mRNA and protein levels in TNBC. *UCHL1* knockdown slows the proliferation of TNBC cells and sensitizes the tumor cells to Tamoxifen and Fulvestrant. *KLF5* overexpression partially reverses these trends. Both TET1 and TET3 can bind to the UCHL1 promoter region, reducing methylation of associated CpG sites and enhancing *UCHL1* transcription in TNBC cell lines. Additionally, TET1 and TET3 elevates KLF5 protein level in a UCHL1-dependent manner.

**Conclusion:**

UCHL1 plays a pivotal role in TNBC by deubiquitinating and stabilizing KLF5, contributing to endocrine therapy resistance. TET1 and TET3 promote *UCHL1* transcription through promoter demethylation and maintain KLF5 protein level in a UCHL1-dependent manner, implying their potential as therapeutic targets in TNBC.

**Supplementary Information:**

The online version contains supplementary material available at 10.1186/s13058-024-01800-1.

## Introduction

Breast cancer has the highest incidence and mortality rates among women globally [1]. It is characterized by molecular diversity and significant heterogeneity [2, 3]. Breast tumors are commonly classified into four subtypes based on the expression of estrogen receptor (ER), progesterone receptor (PR), and human epidermal growth factor receptor 2 (HER2/ERBB2). These subtypes include luminal A; luminal B; HER2 overexpression subtype and triple-negative breast cancer (TNBC) (ER-, PR-, and HER2-). Notably, around 80% of TNBC cases are basal-like [4]. While endocrine therapies such as Tamoxifen are generally effective for ER + breast cancers, approximately 33% of metastatic ER + patients may develop resistance, often due to lost ER expression [5]. ER-negative cancers are more invasive than their ER-positive counterparts, with TNBC being the most aggressive and invasive subtype. The lack of ER, PR, and HER2 expression in TNBC complicates treatment, severely limiting therapeutic options for metastatic cases. Patients ineligible for surgery are often relegated to radiotherapy and chemotherapy, which remain primary contributors to breast cancer-related mortality. Therefore, understanding the mechanisms underlying ER dysregulation in breast cancer and developing tailored therapeutic strategies are crucial for improving survival rates in affected patients.

Ubiquitination is a reversible post-translational modification that significantly influences protein function through the addition of a ubiquitin (Ub) moiety [6]. The process of polyubiquitination, which often leads to proteasomal degradation, has emerged as a critical target in drug development for a variety of diseases [6, 7]. Deubiquitinating enzymes (DUBs), a class of enzymes capable of hydrolyzing ubiquitin, reverse this modification by cleaving the peptide bond between ubiquitin’s C-terminal glycine and the lysine residue on the substrate protein. Currently, around 100 DUBs have been identified, each with specific roles in cellular processes. Among these DUBs, Ubiquitin carboxyl-terminal hydrolase L1 (UCHL1) has garnered attention due to its elevated expression levels in triple-negative breast cancer (TNBC) when compared to other breast cancer subtypes such as Luminal A, Luminal B, and HER2+ [8, 9].

ER can undergo polyubiquitination and subsequent degradation via the proteasomal pathway [10]. UCHL1, through its deubiquitinating activity, stabilizes the epidermal growth factor receptor (EGFR), leading to the overactivation of the MAPK signaling pathway and the suppression of ERα transcription [11, 12]. A significant association has been identified between high *UCHL1* expression and poor prognosis in ER + breast cancer patients receiving Tamoxifen treatment [11]. Additionally, *UCHL1* knockdown markedly increases ER transcription in ER-negative cell lines treated with estrogen, restoring functional activity and reestablishing sensitivity to antiestrogen therapy [11]. Therefore, targeting UCHL1 may restore ER levels in ER-negative breast cancer and TNBC patients and make these breast cancer patients sensitive to endocrine therapy again.

Given the established regulatory role of UCHL1, it is reasonable to anticipate that targeting UCHL1 could enhance the effectiveness of endocrine therapy in ER-negative breast cancer patients and diminish migration and metastasis in TNBC. A number of small molecule inhibitors targeting UCHL1’s enzymatic activity have undergone preclinical evaluation [8, 13]. Therefore, elucidating the mechanisms behind UCHL1 dysregulation is expected to significantly enhance the clinical efficacy of UCHL1 inhibitors. This study is dedicated to investigating the substrates of UCHL1 that are pertinent to endocrine therapy responses and unraveling the pathways of *UCHL1* dysregulation in TNBC.

## Materials and methods

### Bioinformatic data analysis

The pan-cancer dataset (TCGA TARGET GTEx (PANCAN, *N* = 19,131, G = 60,499) that integrated data from the Cancer Genome Atlas (TCGA) and Genotype-Tissue Expression Project (GTEx) were downloaded from the UCSC database (https://xenabrowser.net/) [14]. Then, *UCHL1*, *DNMT1, DNMT3A/B*, and *TET1/2/3* expression in different PAM50 subtypes of breast cancers (primary tumors), and corresponding normal tissues (adjacent normal from TCGA and normal tissues from healthy controls from GTEx were compared. The expression values have been further transformed using log_2_(TPM + 0.001). DNA methylation data (methylation 450k) from this dataset was also extracted. The correlations between gene expression and the β values of the CpGs were assessed by calculating the Pearson’s correlation coefficients.

One recent publication that provided a single-cell and spatially resolved transcriptomics analysis of human breast cancers was analyzed using the Single Cell Portal (https://portals.broadinstitute.org/single_cell) [3].

### Cell culture and treatment

MCF-7 cells (representing Luminal A), BT474 cells (representing Luminal B), SUM149, and MDA-MB-468 cells (representing Basal-like and TNBC) were procured from Procell (Wuhan, Hubei, China). MCF-7 cells were grown in Minimum Essential Medium containing NEAA, 10 µg/mL Insulin, 10% fetal bovine serum (FBS), and 1% P/S (100 U/mL Penicilium and 100 µg/mL Streptomycin). BT474 cells were grown in RPMI-1640 medium supplemented with 10 µg/ml Insulin, 2mM L-glutamine, 20% FBS, and 1% P/S. SUM149 and MDA-MB-468 cells were cultured in Dulbecco’s Modified Eagle Medium (DMEM) with 10% FBS and 1% P/S. All cells were maintained in a cell incubator with 5% CO_2_ at 37℃.

5-Aza-2’-deoxycytidine (5-Aza-dc), cycloheximide (CHX), MG132 Tamoxifen and Fulvestrant were purchased from MedChemExpress (Monmouth Junction, NJ, USA). To induce hypomethylation, cells were treated with 5-Aza-dc (1 µM) for 48 h.

Lentiviral shRNAs were constructed using the pLKO.1-puro plasmids. The following shRNA sequences were used: shUCHL1#1: 5’-CGGGTAGATGACAAGGTGAAT-3’; sh UCHL1#2: 5’-CCAGCATGAGAACTTCAGGAA-3’; shTET1#1: 5’-CCTCCAGTCTTAATAAGGTTA-3’; shTET1#2: 5’- CCCAGAAGATTTAGAATTGAT-3’; shTET1#3: 5’- GCAGCTAATGAAGGTCCAGAA-3’; shTET2#1: 5’-GCGTTTATCCAGAATTAGCAA-3’; shTET2#2: 5’-CCTCAAGCATAACCCACCAAT-3’; shTET2#3: 5’-GCCAAGTCATTATTTGACCAT-3’; shTET3#1: 5’-ACTCCTACCACTCCTACTATG-3’; shTET3#2: 5’-GCCGAAGCTGTGTCCTCTTAT-3’; shTET3#3: 5’-GAACCTTCTCTTGCGCTATTT-3’; scramble control: 5’- CCTAAGGTTAAGTCGCCCTCG-3’. Wide-type lentiviral *UCHL1* (NM_004181) overexpressing particles and the mutant (C90S), Myc-tagged *UCHL1* (Myc-UCHL1), C-terminal 3xFlag tagged full-length *KLF5* (NM_001730) and the truncated fragments (encoding aa1-312 or aa313-457) were generated using pLV-Puro or pLVX-IRES-Puro-3xFlag.

The pMD2.G and psPAX2 vectors were co-transfected with plasmid DNA into HEK 293T cells using TurboFect (Thermo Fisher Scientific Waltham, MA) at a ratio of 5:2:3 to produce lentivirus. After 72 h, the supernatant was collected, filtered through a 0.45 μm filter, and then centrifuged at 25,000 rpm at 4 °C for 1.5 h. The supernatant was then discarded, and the virus pellet was resuspended in an appropriate virus storage solution and left overnight at 4 °C. The packaged virus was collected, and virus titering and specificity testing were performed. The packaged virus was stored at -80℃ until use. Cells were exposed to lentiviral infection at a multiplicity of infection (MOI) of 10, with 6 µg/ml polybrene present.

### Immunofluorescent staining

Immunofluorescent staining was performed following a standard protocol [15]. MDA-MB-468 and SUM149 cells were grown on coverslips. After fixation, permeabilization and blocking, cells were incubated with rabbit anti-KLF5 (1:250, 21017-1-AP, Proteintech, Wuhan, China) and mouse anti-UCHL1 (1:250, 66230-1-Ig, Proteintech). Alexa Fluor 488 labeled secondary anti-rabbit IgG (1: 1000, srbAF488-1, Proteintech) and Alexa Fluor 647 labeled secondary anti-mouse IgG (1: 1000, sms2bAF647-1, Proteintech) were used. Nuclei were counterstained using 4,6-Diamidino-2-phenylindole (DAPI). Immunofluorescent images were captured using IX83 (Tokyo, Japan.

### Co-immunoprecipitation

MDA-MB-468 and SUM149 cells were plated in 10-cm plates for endogenous UCHL1 and KLF5 protein interaction analysis and ubiquitination studies. For UCHL1 and KLF5 protein interaction analysis, cell samples were lysed using cell lysis buffer for Western and IP (P0013, Beyotime, Shanghai, China). For ubiquitination studies, cell samples were lysed using RIPA lysis buffer (P0013B, Beyotime), which contains 0.1%SDS and 1% sodium deoxycholate (two robust anionic detergents) that disrupt the non-covalent interactions holding protein complexes together. These ingredients and formation are appropriate co-immunoprecipitation assays for ubiquitin detection according to previous publications [16–18]. Endogenous proteins were immunoprecipitated with rabbit anti-UCHL1 (21017-1-AP, Proteintech), anti-KLF5 (14730-1-AP, Proteintech) or anti-EGFR (18986-1-AP, Proteintech) antibody respectively, for 120 h at 4.0 °C. Rabbit IgG serves as the negative control. For exogenous FLAG tagged proteins, immunoprecipitation was performed using rabbit anti-Flag (80010-1-RR, Proteintech). 100 µl Protein A Sepharose bead slurry were added to the sample to capture the immunocomplex for 4 h at 4 °C with gentle agitation. Then, the samples were centrifuged at 220G for 30 s at 4 °C to discard the supernatant. After washing with 0.2%TBST, the immunocomplex was eluted for western blotting assays. Mouse derived primary antibodies were applied for subsequent detection to avoid IgG bands.

### Quantitative reverse transcription PCR (RT-qPCR)

RT-qPCR was performed following the method described [19]. In brief, total RNA was isolated from cell, reversely transcribed into cDNA and utilized for template for qPCR. Relative gene expression was normalized with *GAPDH* and calculated using the 2^−ΔΔCT^ method. Amplification was carried out using the following primers: *FGFBP1*, forward: 5’-TGGCAAACCAGAGGAAGACTGC-3’; reverse: 5’-GGAACCCGTTCTCTTTTGACCTC-3’; *EGFR*, forward: 5’-AACACCCTGGTCTGGAAGTACG-3’; reverse: 5’-TCGTTGGACAGCCTTCAAGACC-3’; *ESR1*, forward: 5’- GCTTACTGACCAACCTGGCAGA-3’; reverse: 5’- GGATCTCTAGCCAGGCACATTC-3’; *TET1*, forward: 5’-CAGGACCAAGTGTTGCTGCTGT-3’; reverse: 5’- GACACCCATGAGAGCTTTTCCC-3’; *UCHL1*, forward: 5’- CAGTTCAGAGGACACCCTGCTG-3’; reverse: 5’- CCACAGAGCATTAGGCTGCCTT-3’; *GAPDH*, forward: 5’-GTCTCCTCTGACTTCAACAGCG-3’; reverse: 5’-ACCACCCTGTTGCTGTAGCCAA-3’.

### Western blotting assays

Conventional western blotting was performed following a standard protocol [20]. The following antibodies and dilutions were applied: anti-UCHL1 (1: 1000, 14730-1-AP/66230-1-Ig, Proteintech), anti-KLF5 (1:1000, 66850-1-Ig/21017-1-AP, Proteintech), anti-Ubiquitin (anti-Ub, 1:1000, 10201-2-AP, Proteintech), anti-Flag tag (1:1000, 66008-4-Ig, Proteintech), anti-Myc tag (1:1000, 60003-2-Ig, Proteintech), anti-TET1 (1:1000, 61,443, Proteintech, Wuhan, China), anti-TET3 (1: 1000, ABE290, Merck, Darmstadt, Germany) and anti-β-actin (1:2000, 20536-1-AP, Proteintech). Then, the protein bands were visualized using Enhanced chemiluminescence (ECL) (BeyoECL Star, Beyotime, Shanghai, China).

### Gene set enrichment analysis (GSEA)

For GSEA, we obtained the GSEA software (version 3.0) from the GSEA website [21]. Primary basal-like tumor cases in TCGA-BRCA were divided into high expression ( > = 50%) and low expression groups (< 50%) based on the expression levels of *UCHL1* or *KLF5*. The h.all.v7.4.symbols.gmt gene set collection from the Molecular Signatures Database [22] was to evaluate the related pathways and molecular mechanisms. The gene expression profiles and phenotype groupings were set with a minimum gene set size of 5 and a maximum of 5000, with 1000 permutations. Gene sets with *p*-values of < 0.05 were considered statistically significant.

### Bisulfite sequencing PCR (BSP)

BSP assay was performed following a protocol introduced previously [23]. To summarize, the cell samples were subjected to genomic DNA extraction and then treated with sodium bisulfite using the EZ DNA Methylation-Gold kit (Zymo Research, Irvine, CA, USA). The converted DNA was utilized for PCR assays with bisulfite-specific primers (forward: 5’-TAAAATTAAAGATTTTATTAAAAGGATTGT-3’; reverse: 5’-AAAAAAAACAAAAACAAAACCAAAC-3’), which included 15 CpGs in the PCR products. Afterwards, the QIAquick PCR purification kit from QIAGEN in Germany was used to purify the PCR products, which were then cloned into the pGEM-T Easy Vector from Promega in Madison, WI, USA. Subsequently, five bacterial clones that contained the insert were chosen for sequencing. The position of the primers was marked in Supplementary Fig. 1.

### Chromatin immunoprecipitation (ChIP)-qPCR

ChIP was performed with a commercial ChIP Assay Kit (Beyotime) [24]. MDA-MB-468 and SUM149 cells with or without lentivirus-mediated *TET1* or *TET3* knockdown were collected and lysed. Immunoprecipitation was performed using anti-TET1 (61443, Proteintech), anti-TET3 (ABE290, Merck) or rabbit IgG (negative control). Then, the immunoprecipitated chromatin samples were purified and used for qPCR assays. The following primers were used: F:5’-ACCGGCGAGTGAGACTG-3’ and R:5’-CACTGTGAGGCCTGTGC-3′. The position of the primers was marked in Supplementary Fig. 1.

### Colony formation

MDA-MB-468 cells with *TET1* or *TET3* knockdown alone or combined with *UCHL1* overexpression were placed into 24-well plates (500 cells per well). Cells were cultured for 14 days with or without Tamoxifen (10 µM) or Fulvestrant (500 nM) treatment. Then, the colonies were fixed, stained, and counted.

### Animal studies

Animal studies were conducted following a protocol introduced previously [19]. The animal study was carried out at Jinruijie Biotechnology Service Center in Chengdu, China, and received approval from the institution’s ethics committee (Approval no. 2023056SPPH). All procedures involving animals adhered to the Guide for the Care and Use of Laboratory Animals [25]. Female athymic nude mice (BALB/c-nu), weighing approximately 18–20 g and aged 5–6 weeks, were procured from Vital River Laboratory Animal Technology (Beijing, China). These mice were raised in a specific-pathogen-free (SPF) environment. To initiate the experiment, 5 × 10^6^ MDA-MB-468 cells (with indicating treatment) in a mixture of 0.2 ml PBS and Matrigel matrix (BD Biosciences) in a 1:1 volume ratio were injected subcutaneously into the fourth mammary fat pad. Tumor volume was calculated as length × width^2^× (π/6). After the tumors became palpable, the mice were randomly assigned to one of four groups (*n* = 6/group). Once the mean diameter of the tumors reached 5–6 mm, the mice were administered the specified treatment (1 mg per dose via oral gavage daily or vehicle control) for 18 days.

The tumor sizes and body weights of mice were measured every other day until euthanization (on the same day using CO_2_ asphyxiation). Before euthanization, to obtain the peripheral blood sample, a heparin blood collection tube was used, and the supernatant was collected after centrifugation. The collected peripheral blood sample was then analyzed using an automatic biochemical analyzer (Mindray, China) to determine the concentrations of ALT, AST, creatinine, and urea. Then, the xenograft tumors were excised and utilized for IHC staining of Ki-67 and ERα.

### Statistical analysis

GraphPad 8.01 was used to collect and integrate the statistical results. The quantitative data was presented as mean ± SD. To compare two groups, an unpaired T-test was used. Pearson’s r or Spearman’s rho were utilized to assess correlation. To evaluate the differences in multiple groups, one-way ANOVA with post-hoc Tukey’s multiple comparisons test was performed. Significance was set at *p* < 0.05.

## Results

### UCHL1 stabilizes KLF5 via deubiquitination in TNBC cells

**Fig. 1:**
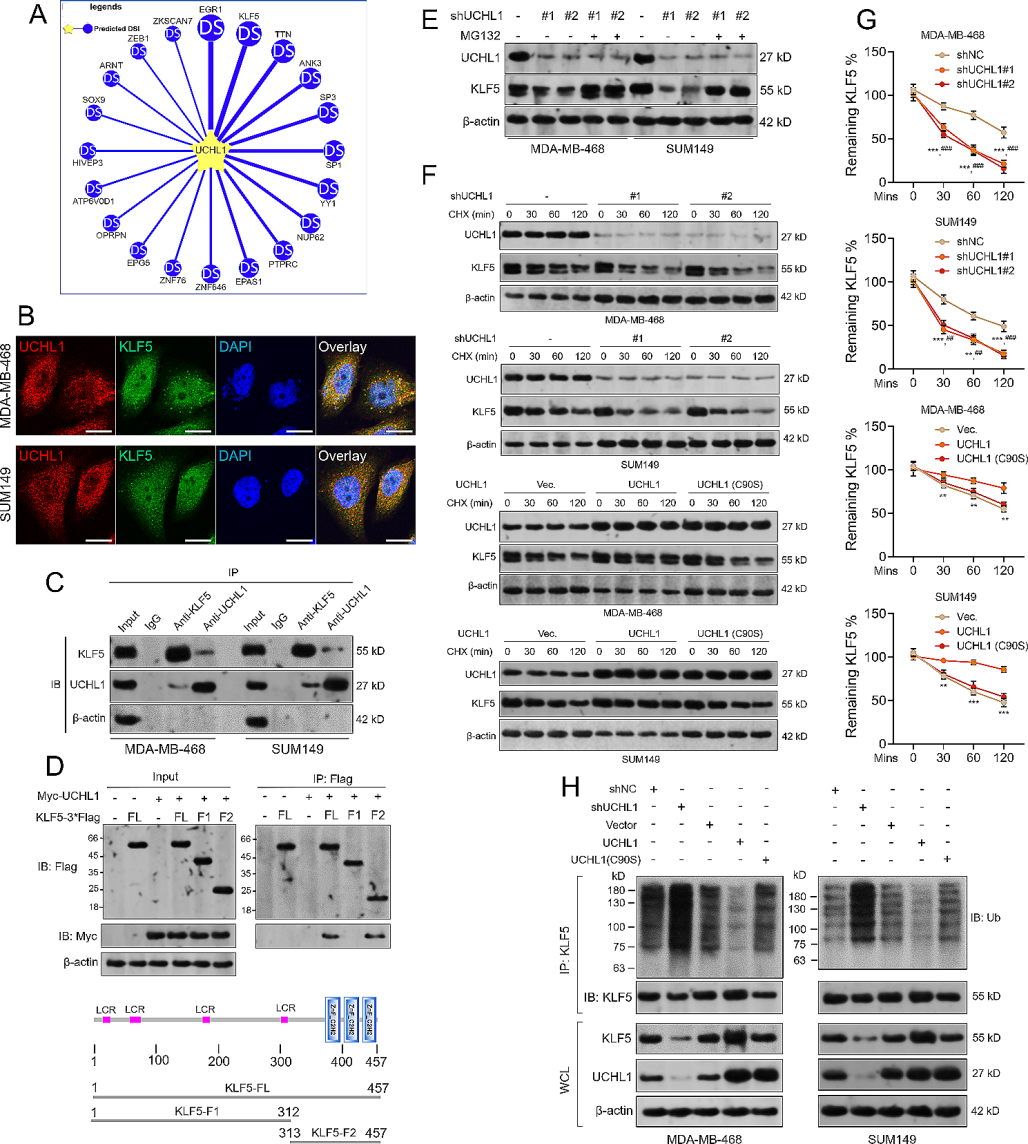
UCHL1 stabilizes KLF5 via deubiquitination in TNBC cells. **(A)** Predicted interactions between UCHL1 and its potential deubiquitinase-substrates, using UbiBrowser 2.0. **(B)** Immunofluorescence staining was used to detect the subcellular location of UCHL1 and KLF5. 4,6-Diamidino-2-phenylindole (DAPI) was used to stain the DNA. Scale bar, 10 µM. **(C)** Co-immunoprecipitation was performed to detect endogenous interactions between UCHL1 and KLF5 proteins in MDA-MB-468 and SUM149 cells. Endogenous proteins were immunoprecipitated with the rabbit anti-KLF5 or anti-UCHL1 antibody. Rabbit IgG serves as the negative control. The presence of immunoprecipitated proteins were detected using mouse anti-UCHL1 or anti-KLF5 antibody respectively. The experiment was repeated three times, and a representative result is shown. **(D)** Mapping the KLF5 domain that interacts with UCHL1. FLAG-tagged full-length or mutants of KLF5 (a schematic diagram is shown below the panel) and Myc-UCHL1 were co-transfected into MDA-MB-468 cells. Immunoprecipitation was performed with FLAG-M2 beads. The experiment was repeated three times, and a representative result is shown. **(E)** The expression of UCHL1 and KLF5 protein in MDA-MB-468 and SUM149 cells with *UCHL1* knockdown, with or without the presence of MG132 (10 µM for 4 h). **F-G.** UCHL1 positively modulates the half-life of KLF5. MDA-MB-468 or SUM149 cells were infected for UCHL1 knockdown (F, top two panels) or overexpression (F, bottom two panels) and treated with cycloheximide (CHX) for the indicated time points. Western blotting was used to measure the endogenous KLF5 protein level. **G.** The graphs showing the quantitative results of KLF5 levels in panel F by ImageJ. Error bars represent S.D. The experiment was repeated three times, and a representative result is shown. *, comparison between shNC and shUCHL1#1 or between vector and UCHL1; #, comparison between shNC and shUCHL1#2. **H.** UCHL1 modulates endogenous KLF5 protein ubiquitination in TNBC cells. MDA-MB-468 (left) and SUM149 (right) cells were infected for *UCHL1* knockdown or overexpression (wild-type or C90S) for 48 h. Scramble shRNA or vector were used as the negative control. After the cells were treated with MG132 (10 µM for 4 h), cells were lysed for immunoprecipitation using anti-KLF5. Immunoblotting was used to detect the ubiquitinated KLF5 protein levels. The experiment was repeated three times, and a representative result is shown. IP, immunoprecipitation; IB, immunoblotting. DSI: deubiquitinase-substrate interactions. LCR: low complexity region. ** and ^##^, *p* < 0.01; *** and ^###^, *p* < 0.001

UCHL1 is a deubiquitinating enzyme known for its role in catalyzing the deubiquitination of multiple substrates. Utilizing the UbiBrowser 2.0 (http://ubibrowser.bio-it.cn/ubibrowser_v3/) [26], we identified the top 20 high-potential substrates of UCHL1 (Fig. 1A). Notably, KLF5 emerged as a putative substrate with significant links to breast cancer pathology [27–29]. Three DUBs, including ATXN3L, BAP1, and USP3 can deubiquitinate and stabilize KLF5 that are implicated in the pathological development of breast cancer [24, 35, 36]. Using data from Cancer Cell Line Encyclopedia (CCLE) database (https://sites.broadinstitute.org/ccle/datasets) [30], we confirmed positive expression of BAP1, USP3 and UCHL1 in two TNBC representative cell lines, MDA-MB-468 and SUM149 (Supplementary Fig. 2A).

In principle, UCHL1 and KLF5 proteins are supposed to interact mutually if UCHL1 is a KLF5 deubiquitinating enzyme. Immunofluorescent staining confirmed the co-localization between UCHL1 and KLF5 in the cytoplasm of MDA-MB-468 and SUM149 cells (Fig. 1B). Subsequent co-IP assays using endogenous proteins confirmed the mutual interactions between UCHL1 and KLF5 (Fig. 1C). To characterize the domain of KLF5 required for the interaction, we generated two KLF5 fragments, including KLF5-FL1 (1-312), which mainly contains four low complexity regions (LCR) and KLF5-FL2 (313–457) that contains three zinc finger domains. Co-IP assays showed that the full-length KLF5 and KLF5-FL2 can interact with UCHL1 but not KLF5-FL1 (Fig. 1D). These results imply that the N-terminal domains of KLF5 are pivotal for its interaction with UCHL1.

To further explore whether UCHL1 modulates KLF5 protein stability, we knocked UCHL1 down in MDA-MB-468 and SUM149 cells (Fig. 1E). However, when MG132 was administrated to prevent proteasomal degradation, *UCHL1* knockdown-mediated KLF5 downregulation was largely canceled (Fig. 1E). A reduction in endogenous KLF5 levels was observed following the knockdown of *UCHL1* (Fig. 1E). Subsequent fractionation western blotting confirmed that *UCHL1* knockdown induced nuclear KLF5 downregulation (Supplementary Fig. 2B). However, *UCHL1* knockdown did not alter *KLF5* transcription (Supplementary Fig. 2C). These findings imply that UCHL1 might alleviate KLF5 degradation via the proteasomal pathway. Then, the effect of UCHL1 on the half-life of the KLF5 protein was then quantified using a cycloheximide (CHX) chase assay. Compared with the control, the KLF5 protein half-life was shortened in MDA-MB-468 and SUM149 cells when UCHL1 was knocked down (Fig. 1F-G). In addition, we observed that the KLF5 protein half-life was prolonged by wild-type UCHL1 overexpression but not by UCHL1 C90S (Fig. 1F-G), a deubiquitination activity-deficient mutant [31, 32].

To test whether UCHL1 increases endogenous KLF5 protein stability via deubiquitination, MDA-MB-468 and SUM169 cells with *UCHL1* knockdown or overexpression (wild-type or C90S) were subjected to co-IP assays to detect KLF5-specific ubiquitination. UCHL1 (C90S) can still bind to KLF5 (Supplementary Fig. 3A). This study relied on the endogenous ubiquitin present in the cells. Therefore, MG132 was used to prevent the degradation of ubiquitinated proteins, allowing us to detect ubiquitination events more reliably. When MG132 was not administered, in ubiquitination-related smear band was observed (Supplementary Fig. 3B). Compared to the control cells, *UCHL1* knockdown remarkably increased the endogenous KLF5 protein polyubiquitination. In comparison, Wild-type UCHL1 overexpression, but not the C90S mutant, decreases KLF5 protein ubiquitination (Fig. 1H). No ubiquitination-specific smear bands were observed in IgG control groups (Supplementary Fig. 3C). These results suggest that UCHL1 interacts and stabilizes KLF5 by reducing its polyubiquitination.

### UCHL1 negatively regulates ERα expression and the responses to endocrine therapy via KLF5 in TNBC

UCHL1 can increase resistance to endocrine therapy by increasing the degradation of estrogen receptor α (ERα) via EGFR [11]. The deubiquitination effect of UCHL1 on EGFR was validated in MDA-MB-468 and SUM149 cells (Supplementary Fig. 4A). Besides, KLF5 can activate EGFR transcription via promoter binding [33]. Then, we accessed RNA-seq data from the TCGA-BRCA dataset, specifically analyzing basal-like tumor cases due to their substantial overlap with TNBC cases. Subsequently, we performed Gene Set Enrichment Analysis (GSEA) to compare the gene signatures between cases with high versus low expression levels of *UCHL1* and *KLF5*. Results revealed that cases with higher expression of *UCHL1*, as well as those with higher expression of *KLF5*, both showed significant enrichment in gene sets associated with the estrogen response (Supplementary Fig. 4B). These data imply that UCHL1 and KLF5 might act as upstream regulators of ERα expression in TNBC.

**Fig. 2:**
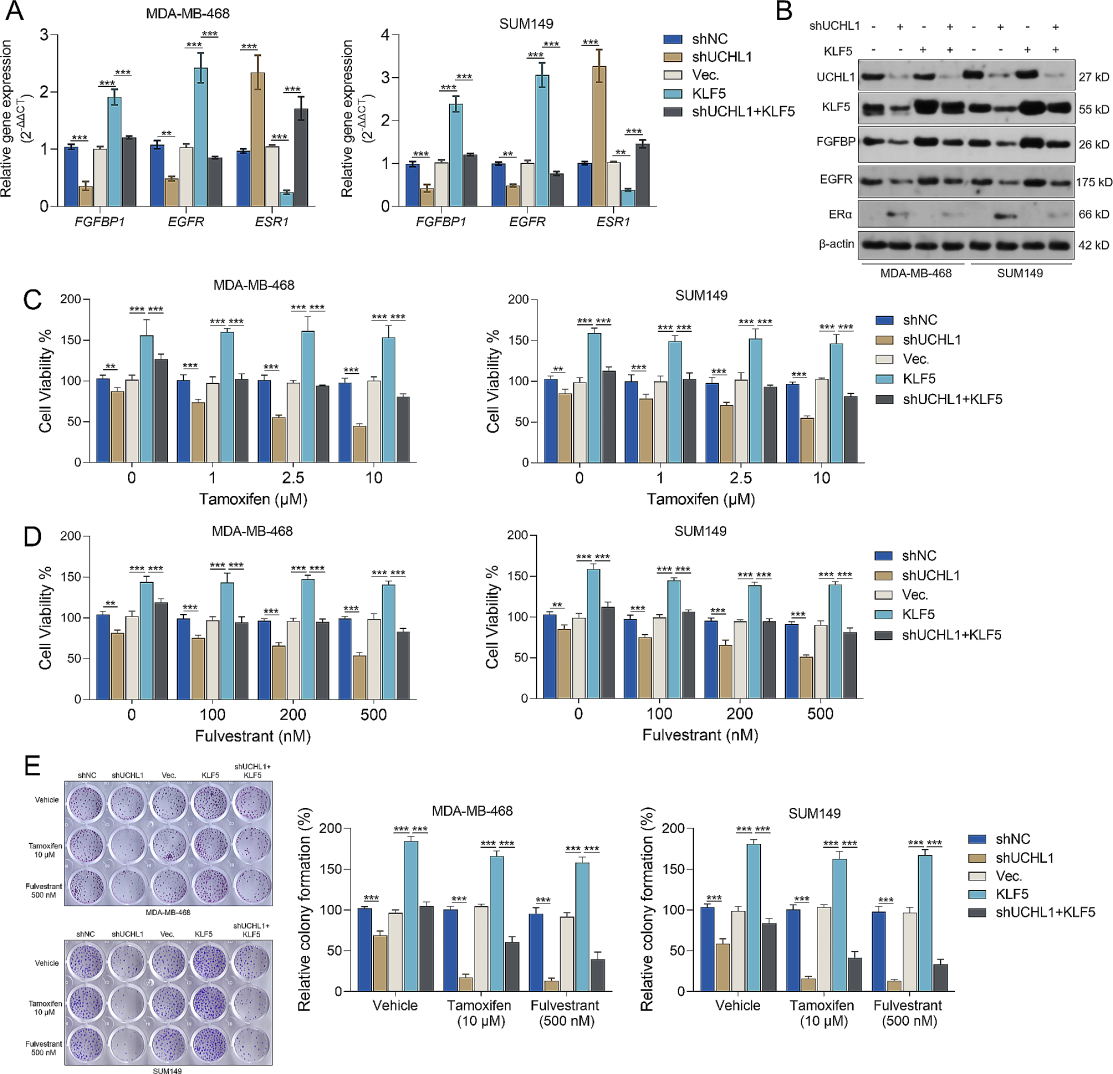
UCHL1 negatively regulates ERα expression and the responses to endocrine therapy via KLF5 in TNBC cells in vitro **A-B.** *FGFBP1, EGFR*, and *ESR1* mRNA (**A**) and protein (**B**) expression in MDA-MB-468 and SUM149 cells with *UCHL1* knockdown, *KLF5* overexpression alone or in combination **C-D**. MDA-MB-468 and SUM149 cells with *UCHL1* knockdown alone or combined with *KLF5* overexpression were treated with Tamoxifen (**C**) or Fulvestrant (**D**) for 72 h. Cell viability was measured using CCK-8 assay. **E**. Colony formation of MDA-MB-468 and SUM149 cells with *UCHL1* knockdown alone or in combination with *KLF5* overexpression were treated with Tamoxifen (10 µM, top panels) or Fulvestrant (500 nM, bottom panels). Results were mean ± SD from three biologically independent experiments. ***, *p* < 0.001

Our qRT-PCR and western blotting results showed that *UCHL1* knockdown decreased *FGFBP1* (a known KLF5 transcriptional target gene [34]) and *EGFR* expression but increased *ESR1* expression at both mRNA and protein levels (Fig. 2A-B, Supplementary Fig. 7). However, these effects were partially reversed by *KLF5* overexpression (Fig. 2A-B, Supplementary Fig. 7). These findings suggest that KLF5 is a downstream effector of UCHL1 in modulating *ESR1* expression in TNBC cells.

Then, we tested how the UCHL1-KLF5 axis modulates endocrine therapeutic responses of TNBC cells in vitro and *in vivo.* While the knockdown of UCHL1 alone does result in a decrease in cell viability, this effect is indeed not pronounced (Fig. 2C-D). Cytotoxicity of Tamoxifen and Fulvestrant against MDA-MB-468 and SUM149 cells was significantly increased when expression of *UCHL1* was knocked down (Fig. 2C-D). Besides, the proliferation of these two cells were also significantly hampered by combining *UCHL1* knockdown and endocrine therapy reagent (Tamoxifen or Fulvestrant) (Fig. 2E). In comparison, *KLF5* overexpression partially reversed these alterations (Fig. 2C-E).

**Fig. 3:**
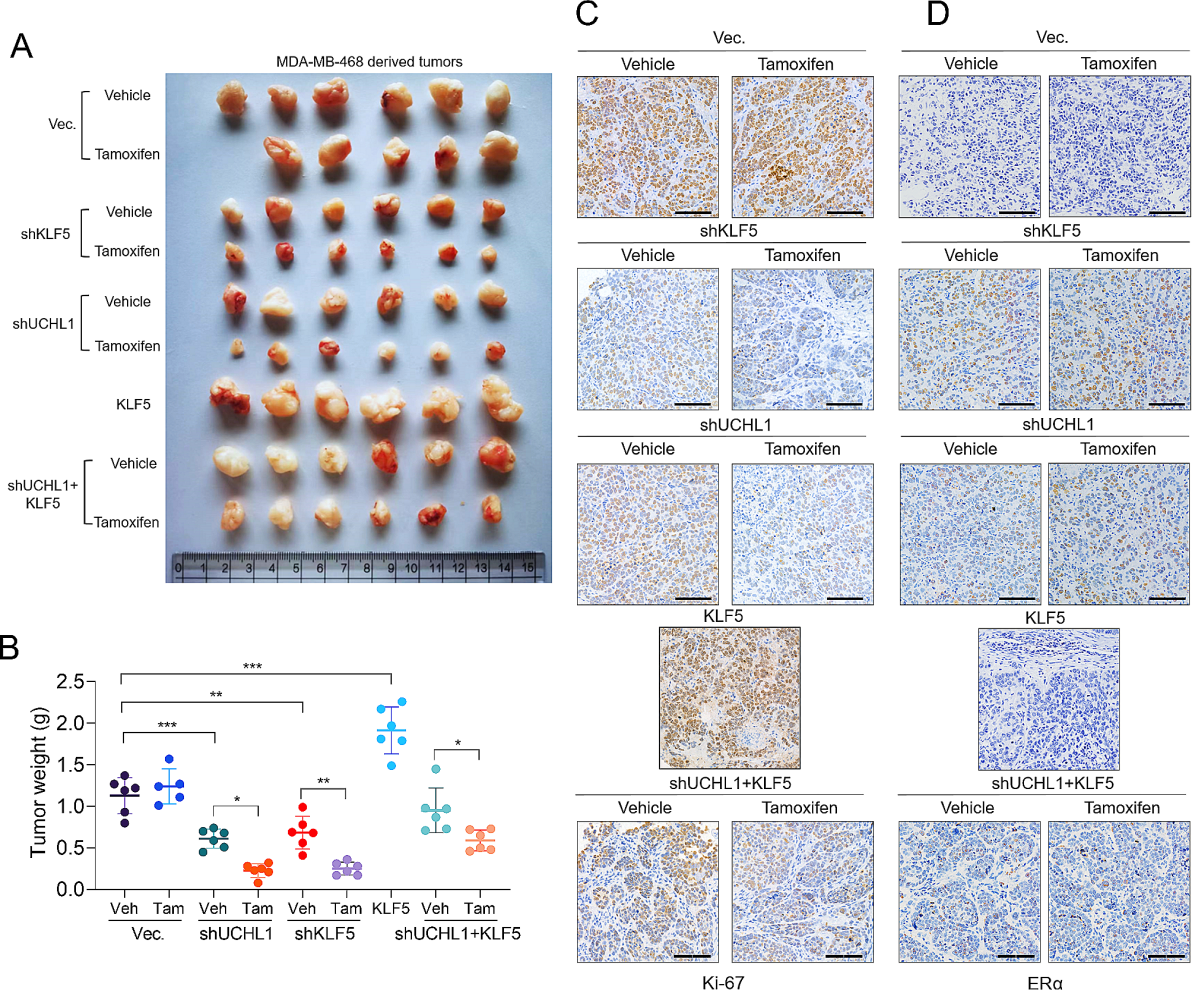
UCHL1 negatively regulates ERα expression and the responses to endocrine therapy via KLF5 in TNBC in vivo. 5-week-old female nude mice were inoculated s.c. with MDA-MB-468 TNBC cells with indicated lentivirus infections. The tumor-bearing mice then received indicated treatment. The tumor sizes were measured on the days as indicated. **(A)** Subcutaneous tumors were excised, and photographs were taken at the termination of the experiment. **(B)** Tumor weight was measured after removal. Data represents the mean ± SD of tumor sizes of each group (*n* = 5 or 6). **C-D.** Immunohistochemistry staining for Ki-67 **(C)** and ERα **(D)** in the tumor specimens from the mice. *, *p* < 0.05; **, *p* < 0.01; ***, *p* < 0.001

For in vivo studies, MDA-MB-468 cells with *UCHL1* knockdown alone or combined with *KLF5* overexpression were subcutaneously injected into nude mice. Subsequently, tumor-bearing animals received Tamoxifen (1 mg per dose via daily oral gavage). In line with our in vitro observations, Tamoxifen treatment did not affect the proliferation of tumors derived from naïve MDA-MB-468 cells. However, knockdown of *UCHL1* or *KLF5* could slow the proliferation of MDA-MB-468 xenograft tumors and also sensitize the tumors to Tamoxifen (Fig. 3A-C), without significant changes in body weight and cytotoxicity to the liver and kidney (Supplementary Fig. 5A-B). In addition, the restoration of ERα proteins in tumors with *UCHL1* or *KLF5* knockdown was observed (Fig. 3D).

### UCHL1 expression in basal breast cancer is negatively correlated with its promoter methylation

**Fig. 4:**
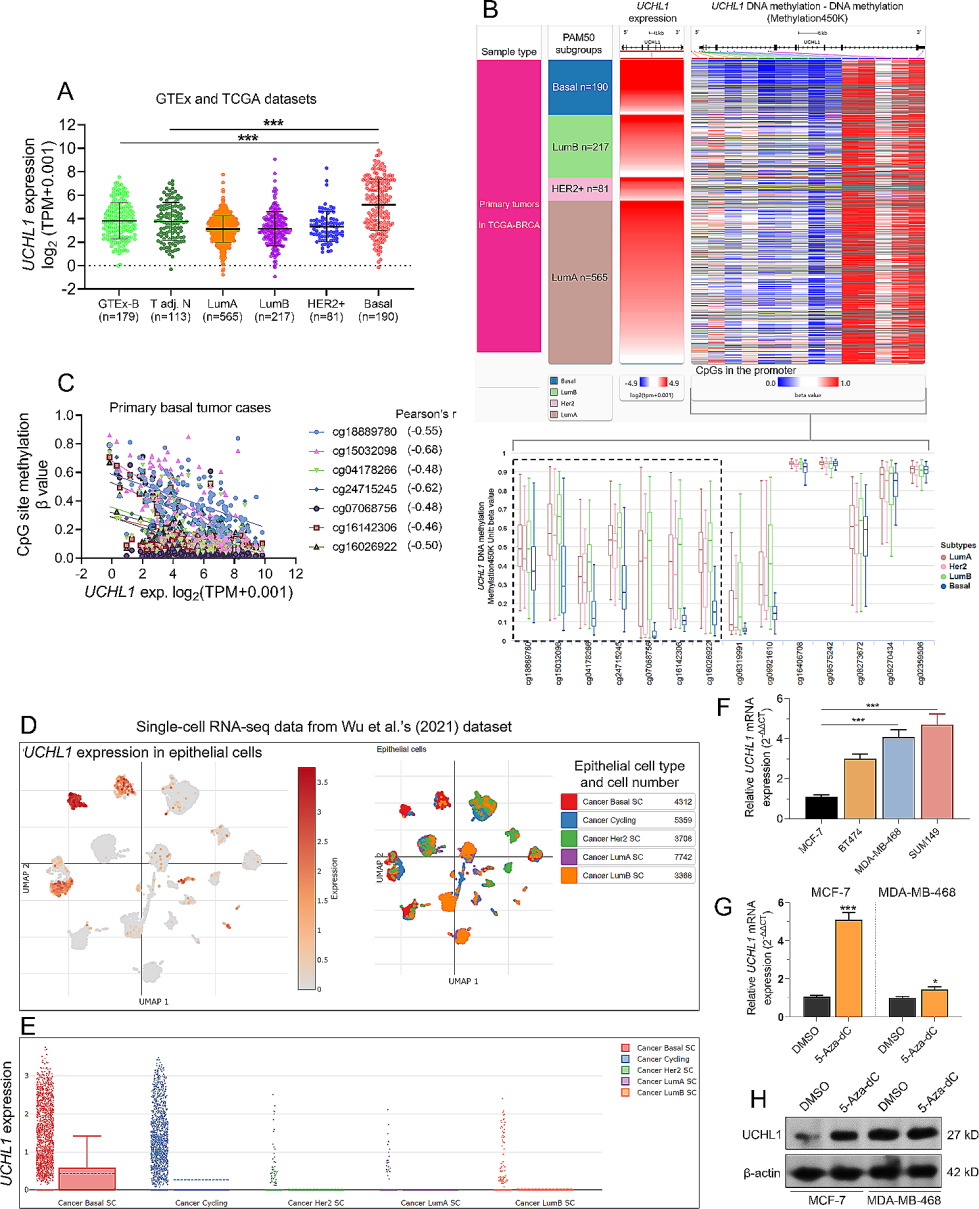
Correlation analysis of *UCHL1* promoter CpG site methylation levels and gene expression in breast cancer tissues. **(A)** Comparison of *UCHL1* expression in normal breast tissue (GTEx-B), TCGA breast cancer (TCGA-BRCA) subtypes, and adjacent cancer tissues (T adj.). **(B)** A heatmap (top) showing the correlation between *UCHL1* expression and *UCHL1* DNA methylation levels in corresponding tissues in TCGA-BRCA subtypes. The black box marks 7 methylation sites related to the promoter region. A bar chart (bottom) comparing *UCHL1* DNA CpG site methylation levels in TCGA-BRCA subtypes. The black box marks 7 methylation sites related to the promoter region. **(C)** Correlation analysis of methylation levels of 7 methylation sites related to the promoter region and *UCHL1* expression levels. **(D)** A UMP plot chart showing the single cells from breast cancer tissues, color-coded by UCHL1 expression (left) and their associated cell type cluster (right). **(E)** A scatter plot illustrates the expression of *UCHL1* in various molecular subtypes of breast cancer and circulating tumor cells. The data is from a recent single-cell sequencing dataset [3], from: https://singlecell.broadinstitute.org/single_cell/study/SCP1039/a-single-cell-and-spatially-resolved-atlas-of-human-breast-cancers. **(F)** qRT-PCR was used to detect the expression of *UCHL1* in MCF-7, BT-474, MDA-MB-468, and SUM149 cells. **G-H.***UCHL* expression in MCF-7 and MDA-MB-468 cells after treatment with the methylation reagent 5-Aza-Dc at the mRNA **(G)** and protein **(H)** levels in MCF-7 and MDA-MB-468 cells. ***, *p* < 0.001

To explore the mechanisms of UCHL1 dysregulation, we merged and compared the expression data of *UCHL1* from the GTEx (normal tissue sequencing data) and TCGA databases (cancer and adjacent tissue). The expression of *UCHL1* in basal-type (mainly TNBC) breast cancer tissue is significantly higher than that in normal breast tissue and adjacent normal tissue (Fig. 4A). The expression of UCHL1 in TNBC is significantly higher than in luminal A/B and HER2 + subtypes (Fig. 1A). Subsequently, we analyzed the correlation between *UCHL1* expression in each subtype of TCGA-BRAC cancer tissue and the corresponding *UCHL1* DNA methylation level in the tissue (Fig. 4B). The methylation levels of seven CpGs (cg18889780, cg15032098, cg04178266, cg24715245, cg07068756, cg16142306, and cg16026922) associated with the *UCHL1* promoter were the lowest in basal type (Fig. 4B). Of the 141 basal-type tumors with sequencing data, 84 had DNA methylation data. Through Pearson correlation analysis, we found a moderate (-0.4 to -0.6) to high (-0.6 to -0.8) negative correlation between the methylation levels of the seven CpGs linked to the *UCHL1* promoter and its gene transcription expression levels (Fig. 4C).

Both TCGA and GTEx are derived from bulk tissue sequencing and represent the comprehensive expression of various cells, so they do not truly reflect the expression of *UCHL1* in tumor cells very well. Then, we analyzed recent single-cell sequencing data in breast cancer [3]. *UCHL1* is mainly expressed in breast tumor cells (Fig. 4D), and its expression in basal-type and circulating tumor cells is significantly higher than that in Luminal A/B and HER2 + subtypes (Fig. 4E). qRT-PCR showed that the expression of *UCHL1* in MDA-MB-468 and SUM149 cells (basal TNBC cell lines) was remarkably higher than that in MCF-7 cells (Luminal A cell line) (Fig. 4F). Treatment with the methylation reagent 5-Aza-dC significantly increased *UCHL1* transcription in MCF-7 and MDA-MB-468 cells (Fig. 4G). However, this change was more pronounced in MCF-7 cells. In addition, treatment with 5-Aza-dC also significantly increased UCHL1 protein expression in MCF-7 cells (Fig. 4H), indicating a consistent trend of UCHL1 expression at the transcriptional and translational levels.

### Bioinformatics analysis identifies TET1 and TET3 as critical demethylation enzymes related to UCHL1 expression

**Fig. 5:**
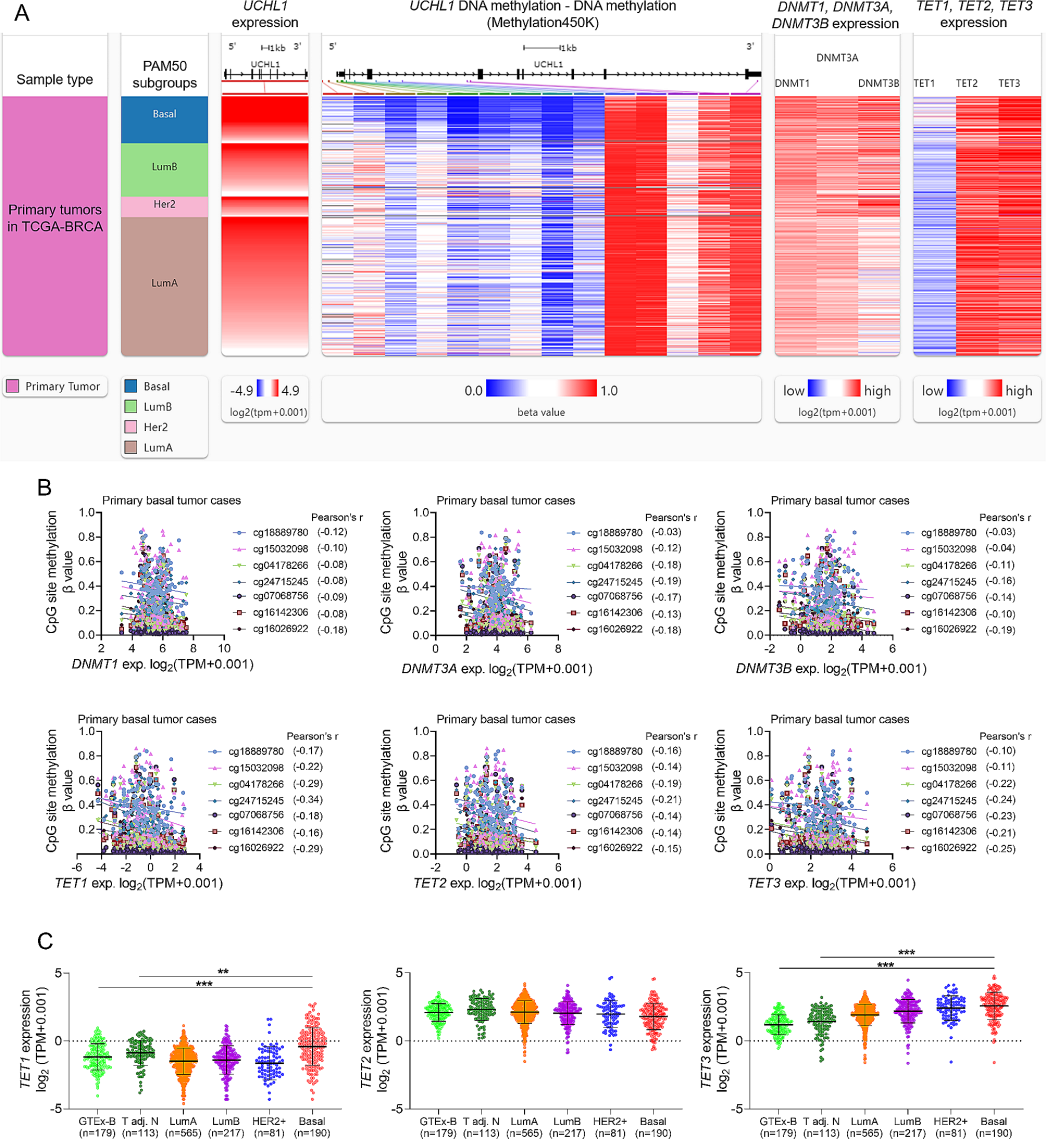
The correlation between *UCHL1* and the expression of genes encoding DNA methyltransferases and demethylases. **(A)** A heatmap showing the correlation among the expression of *UCHL1*, DNA methyltransferase genes (*DNMT1*, *DNMT3A* and *DNMT3B*) as well as demethylase genes (*TET1*, *TET2*, and *TET3*) in the primary tumor samples in TCGA-BRAC. **(B)** Correlation analysis between the methylation levels of 7 CpGs in the *UCHL1* promoter region and the expression levels of DNA methyltransferase genes (DNMT1, DNMT3A, and DNMT3B) as well as demethylase genes (*TET1*, *TET2*, and *TET3*). **(C)** Comparison of *TET1, TET2* and *TET3* expression in normal breast tissue (GTEx-B), TCGA breast cancer (TCGA-BRCA) subtypes, and adjacent cancer tissues (T adj.). **, *p* < 0.01; ***, *p* < 0.001

To clarify whether the downregulation of the methyltransferase genes or the upregulation of the demethylase genes enhanced the transcription of *UCHL1*, we further analyzed the expressional correlation of expression of *UCHL1* with methyltransferase genes and demethylase genes in the primary basal subtype TCGA-BRAC (Fig. 5A). By setting Pearson’s correlation coefficients > 0.2 or < 0.2 as the cutoff, we observed that the expression of methyltransferase genes was not correlated with *UCHL1* expression (Fig. 5B). Among the three demethylases (Fig. 5B), There were negative correlations between *TET1* and the methylation of four promoter-associated CpGs, and between *TET3* and the methylation of five promoter-associated CpGs (Fig. 5B). In addition, *TET1* and *TET3*, but not *TET2*, were significantly upregulated in basal-like tumors than in normal breast and adjacent normal tissues (Fig. 5C).

### TET1 and TET3 bind to the UCHL1 gene promoter and contribute to promoter demethylation

**Fig. 6:**
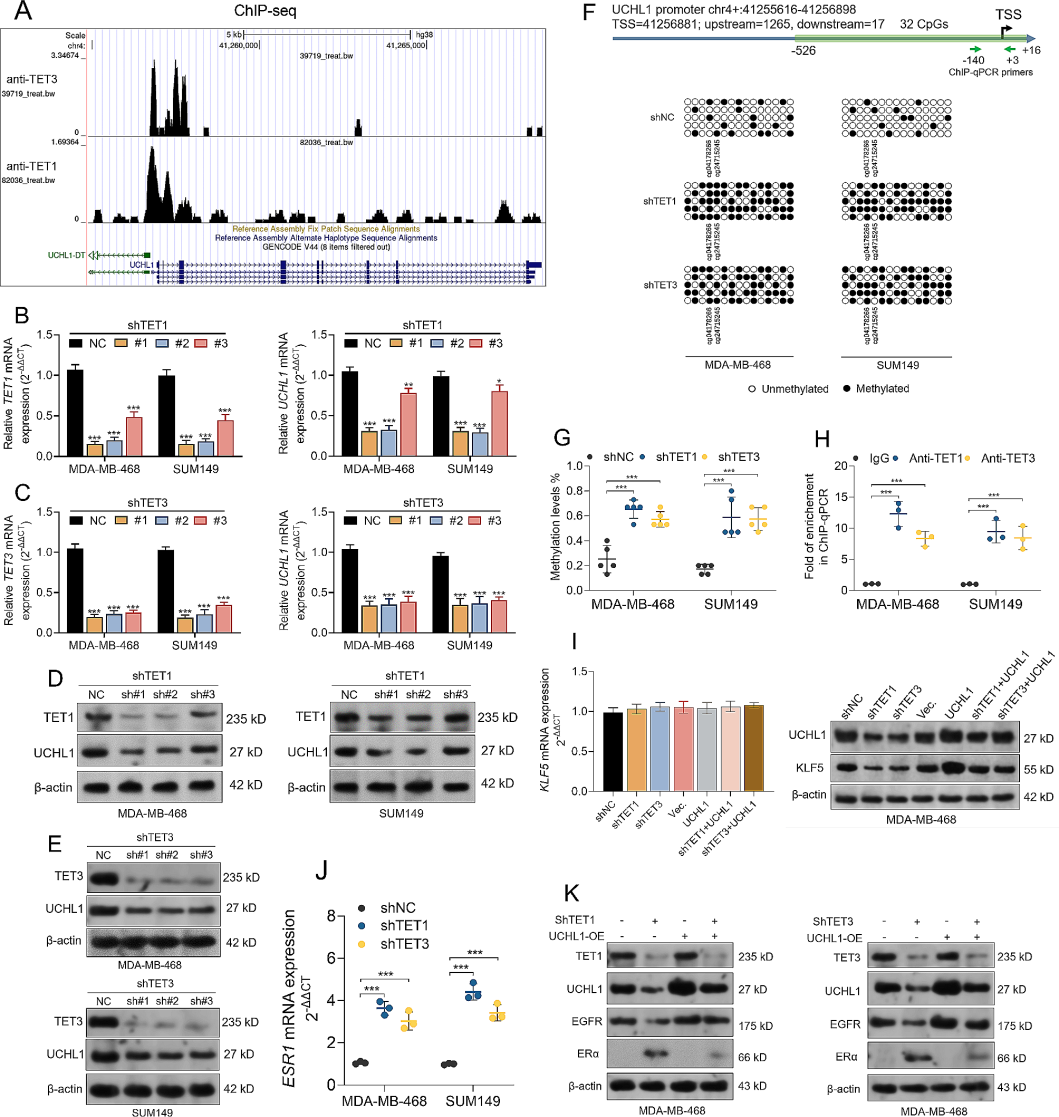
TET1 and TET3 bind to the *UCHL1* gene promoter and contribute to promoter demethylation. **A.** The potential binding sites for TET1 and TET3 in the UCHL1 gene promoter regions. The analysis is based on data from the GSM2642522 and GSM1018960 datasets, which include ChIP-seq data using anti-TET1 or anti-TET3, respectively. **B-E.** The expression of *TET1*, *TET3* and *UCHL1* at both mRNA **(B-C)** and protein **(D-E)** levels in MD-MB-468 and SUM149 cells with *TET1* or *TET3* knockdown. **F-G.** Representative images **(F)** and quantitation **(G)** of BSP assays to detect the methylation of CpGs upon *TET1* or *TET3* knockdown in MDA-MB-468 and SUM149 cells. Data are represented as mean ± SD from three independent experiments. **H.** ChIP-qPCR assay was performed to show the enrichment of *UCHL1* promoter segments in MDA-MB-468 and SUM149 cells with or without *TET1* or *TET3* knockdown. ChIP was conducted using anti-TET1 or anti-TET3, respectively. **I.***KLF5* mRNA (left) and protein (right) expression in MDA-MB-468 cells with *TET1* or *TET3* knockdown alone or in combination with *UCHL1* overexpression. **J**. *ESR1* mRNA expression in MDA-MB-468 and SUM149 cells with TET1 or TET3 knockdown. **K.** EGFR and ERα protein expression in MDA-MB-468 cells with *TET1* or *TET3* knockdown. **, *p* < 0.01; ***, *p* < 0.001

Using ChIP-seq data collected in the CistroDB (http://cistrome.org/db/) [35], we confirmed the potential binding of TET1 and TET3 to the *UCHL1* promoter (Fig. 6A). RT-qPCR and western blotting assays confirmed that knockdown of endogenous *TET1* or *TET3* decreased *UCHL1* expression at the transcriptional and transitional levels in MDA-MB-468 and SUM149 cells (Fig. 6B-E). In comparison, *TET2* knockdown did influence *UCHL1* transcription (Supplementary Fig. 6A-B). BSP assays showed that knockdown of *TET1* or *TET3* substantially elevated the methylation levels within the *UCHL1* promoter (Fig. 6F-G). ChIP-qPCR assays confirmed the enrichment of the *UCHL1* promoter fragments in the anti-TET1 or anti-TET3 immunoprecipitated samples (Fig. 6H). Since we confirmed that TET1 and TET3 can regulate *UCHL1* expression, we further explored whether these two enzymes regulate KLF5 expression via UCHL1. In MDA-MB-468 cells, *TET1* or *TET3* knockdown did not alter *KLF5* transcription but suppress its expression at the protein level (Fig. 6I, Supplementary Fig. 8). The alterations at the protein level were significantly reversed by enforced *UCHL1* overexpression (Fig. 6I, Supplementary Fig. 8). Then, we checked two critical downstream effectors of KLF5, including *EGFR* and *ESR1*. *TET1* or *TET3* knockdown decreased *EGFR* expression but increased *ESR1* expression. However, these effects were weakened by *UCHL1* overexpression (Fig. 6J-K, Supplementary Fig. 9).

## Discussion

*ESR1* transcriptional repression is linked to three primary signaling pathways, including NF-κB, ERK/MAPK, and PI3K [36]. ER-negative breast tumors often exhibit constitutive activation of NF-κB, which inversely correlates with ER expression [36]. Besides, ER-negative breast tumors are frequently associated with EGFR overexpression, which activates MAPK signaling and suppresses *ESR1* transcription [12]. In TNBC, UCHL1 directly interacts with EGFR protein and suppresses EGFR degradation through the ubiquitin-proteasome pathway, leading to suppressed transcription of *ESR1* [11]. Additionally, UCHL1 stabilizes TGFβR1 levels in tumors, acting as an upstream regulator of ERK/MAPK signaling [32, 37]. These alterations collectively drive the development of insensitivity to endocrine therapy. Therefore, inhibiting *UCHL1* expression may provide new treatment options for TNBC. However, as a deubiquitinating enzyme, UCHL1 may be involved in various pro-cancer pathways by stabilizing multiple substrates. Understanding its physiological regulations is a prerequisite for developing pharmaceutical inhibitors.

The current study found that KLF5 is a novel deubiquitination substrate of UCHL1. UHCL1 can interact with KLF5 and reduce its polyubiquitination for proteasomal degradation. KLF5 is a well-characterized transcription factor involved in breast cancer pathology. It activates the expression of *NANOG* and *FGFBP1* in TNBC, which are required for the maintenance of the cancer stem cell (CSC) population [28, 38]. Pharmaceutical inhibition of KLF5 could significantly suppress the CSC properties of TNBC and enhance chemotherapy responses [39, 40]. KLF5 can directly bind to the 5’ regulatory region of *EGFR* and enhance its transcription [33]. Therefore, by maintaining intracellular KLF5 levels, UCHL1 supports *EGFR* expression transcriptionally. Our subsequent analysis showed that inhibiting the UCHL1-KLF5 axis can restore ERα expression and re-sensitize TNBC cells to Tamoxifen and Fulvestrant. These findings imply that the UCHL1-KLF5 axis might be a valuable therapeutic target for TNBC.

Besides UCHL1, some other DUBs, such as ATXN3L, BAP1, and USP3 can deubiquitinate KLF5 and are implicated in the pathological development of breast cancer [29, 41, 42]. Our study contributes to this landscape by identifying UCHL1 as another DUB that stabilizes KLF5 through deubiquitination, thereby enriching the current understanding of the regulatory network governing KLF5 stability. Furthermore, our research complements previous findings [33, 43] by showing that KLF5 can transcriptionally regulate *EGFR* mRNA expression. Unique to our findings, we demonstrate that UCHL1 not only contributes to the regulation of KLF5 but also directly stabilizes EGFR at the protein level through deubiquitination. This dual regulatory capacity of UCHL1, affecting both the mRNA and protein levels of EGFR, distinguishes UCHL1 from other DUBs in the context of breast cancer. It suggests a broader role for UCHL1 in the disease’s molecular pathology, potentially impacting both upstream transcriptional regulation and downstream protein stability.

UCHL1 targeted drugs might be developed by targeting protein activity (small molecule inhibitors) and targeting protein expression (inhibiting its transcription and translation). Therefore, in this study, we also explored the mechanisms underlying the abnormal upregulation of *UCHL1* expression in TNBC. By bioinformatic analysis, we observed that *UCHL1* expression was negatively correlated with the methylation levels of the CpGs in the promoter region. The CpG methylation level in the UCHL1 promoter region in TNBC is significantly lower than that in Luminal A/B and HER2 + tumors. Demethylation treatment increased *UCHL1* expression in breast cancer cell lines. TET1 and TET3 are two potential demethylases of the promoter-related CpGs of *UCHL1*. Both TET1 and TET3 work by starting a series of oxidation reactions that convert the methylated cytosine on the DNA from 5-methyl cytosine (5mC) into 5-hydroxymethylcytosine (5hmC) and further oxidize or convert it into unmethylated cytosine [44, 45]. High *TET1* expression is associated with poor overall survival in TNBC [45]. In addition, its aberrant expression is generally associated with DNA demethylation status in TNBC [45]. TET1 can sustain the activation of the PI3K, EGFR, and PDGF signaling pathways, which are closely associated with TNBC initiation and development [45]. Besides, hypoxia can induce the upregulation of TET1 and TET3 in breast tumor–initiating cells (BTIC), which is required to activate TNFα–p38–MAPK signaling that drives breast tumor malignancy [46]. Our following experiments demonstrated that both TET1 and TET3 can bind to the *UCHL1* promoter region, induce demethylation of the promoter-associated CpGs and enhance the transcription of *UCHL1* in TNBC cell lines. In addition, we observed that knockdown of *TET1* or *TET3* reduced EGFR expression and restored ERα expression in TNBC cells. These findings imply that TET1 and TET3 are two critical enzymes modulating UCHL1 expression and its downstream signaling pathways in TNBC.

## Conclusion

In summary, this study revealed that UCHL1 plays a pivotal role in TNBC by deubiquitinating and stabilizing KLF5, contributing to endocrine therapy resistance. TET1 and TET3 promote *UCHL1* transcription through promoter demethylation and maintain KLF5 protein level in a UCHL1-dependent manner, implying their potential as therapeutic targets in TNBC.

### Electronic supplementary material

Below is the link to the electronic supplementary material.

Supplementary Material 1

Supplementary Material 2

Supplementary Material 3

Supplementary Material 4

Supplementary Material 5

Supplementary Material 6

Supplementary Material 7

Supplementary Material 8

Supplementary Material 9

Supplementary Material 10

## Data Availability

All data are available within the article and its supplementary materials.
