# Supplementary material for: UCHL1 contributes to insensitivity to endocrine therapy in triple-negative breast cancer by deubiquitinating and stabilizing KLF5
Source: Breast Cancer Res. 2024 Mar 11;26:44. doi: 10.1186/s13058-024-01800-1 (PMC10929172; doi:10.1186/s13058-024-01800-1)
Supplement: Supplementary file 1 — Supplementary Material 1 [file 13058_2024_1800_MOESM1_ESM.docx]

>HPRM45359 NM_004181; name=UCHL1; Entrez_ID=7345; Genome=hg38; chr4+:41255616-41256898; TSS=41256881; Upstream=1265, Downstream=17; Length=1283

GGCTCTTGGAGCCCAGTTTAGCAGGGTTTACTTCTCAGTTTTACTTTTCAATTGTGACCGTAAGCAAATTAACTTCTCTAGGCCTGGGATTCTGATCTGTAAAATTGCACTAATATGAGTCTCTTCACGGCTCCTCTAAGGATTAAATGAGAGACACATGCAAAGGATCCCCAAAAACAATAACTCAAAAAATGTTGATTCCCTCCCTTCCCTCTGTCATCTGTTAACCTCAACTTCCTAAATAGAAGGTCTATTCTTTTACCATCATCATTATTCTCTTCGGTGCCTATTTTTAAAAAATACTCAACCTTCTTGCTTCCTTCGCTACCTAAGTATTTCTGCAAGCCCACTTTGTTCTGCAGCTTAGCTTTCCTGGCACAATTCTTATAGATTTTGGTCCCTTTTAAAATTCATTCTTCAGCAAATGCTTTCTCTCTCCATCTTTTGACTAGAGATCATTAGAGATCACCTGAGATCATTAGAGAACAGTGGTTTCCTTGGTTGCCATTCCCTTTCTTCTTCATTGGGAGTATTCTGCGGTGAACTCAGACATTTTATTTTTCAAAGCTTCCCATTCTTTTAAAAATGCTTTTCCTTTTACAGCCTCTCGCTCAAAATCATACCCATCTTTTCCCTGGATCTGTTTTCTCAAGTCTCCAATCGCCTGCCTTCTTTGTGTCTTGTATTACCCTCACATCCCCCAGCTTTCTACTGCTCTCCCAGGACCAACCATTTCTTCCG**CG**GGAGTCACATTACATCAGCATTCCTAATGCAGTATCTGTTATCTACCAGATTCTGTTTTATTCTAGGTAGTCACTTAAAAACGAACCTCGGTACTGGTCTGACTTAACATGGAGGAGGAATTGTCTAAGGTTAAACGCAAACTGCTGAGAGATTTGGGGCGGGGGGCACACATTTACATTCATT**CG**TATTAAATATATACCTGTTGAATTTGTGCTTTTTCTCAAATGCTTCAGAGACTCGAGCTTTAGAGTAATTGGGATGGTGAAAGGATGGGTTTCCAGAAACTTCGCCCAAAATTAAAGACTCCATCAAAAGGACTGCTCCATACACTCAAGGAACACCCACCAACAAATCCCGTCTCCACAACCACCAGATTATCTCACCGGCGAGTGAGACTGCAAGGTTTGGGGGCC**CG**GC**CG**TACCACTCCGCGCTGCGCACGGGGGGTTCGTACCCATCTGGCCGCGACCGTCCGTTTCCCCCTCGCTTGGTTCTGCCCCTGCTCCCCCTGCACAGGCCTCAC**A**GTGCGTCTGGC**CG**GCGC

**A TSS site**

**cg18889780 cg15032098 cg04178266 cg24715245 cg07068756**

ChIP-qPCR primers

BSP primers
