# Supplementary figures and images for: UCHL1 contributes to insensitivity to endocrine therapy in triple-negative breast cancer by deubiquitinating and stabilizing KLF5
Source: Breast Cancer Res. 2024 Mar 11;26:44. doi: 10.1186/s13058-024-01800-1 (PMC10929172; doi:10.1186/s13058-024-01800-1)

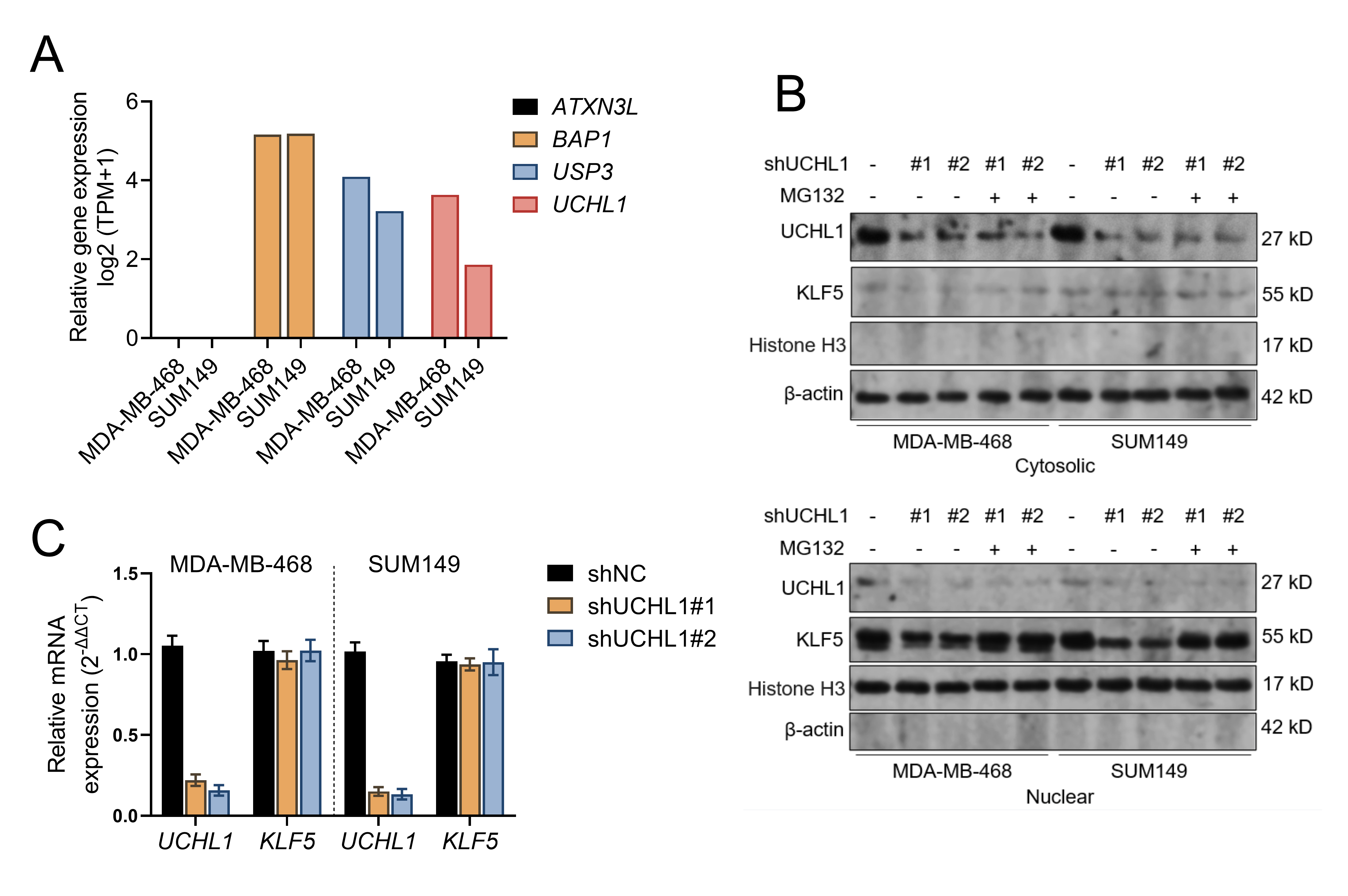

Supplement: Supplementary file 2 — Supplementary Material 2 [file 13058_2024_1800_MOESM2_ESM.tif]

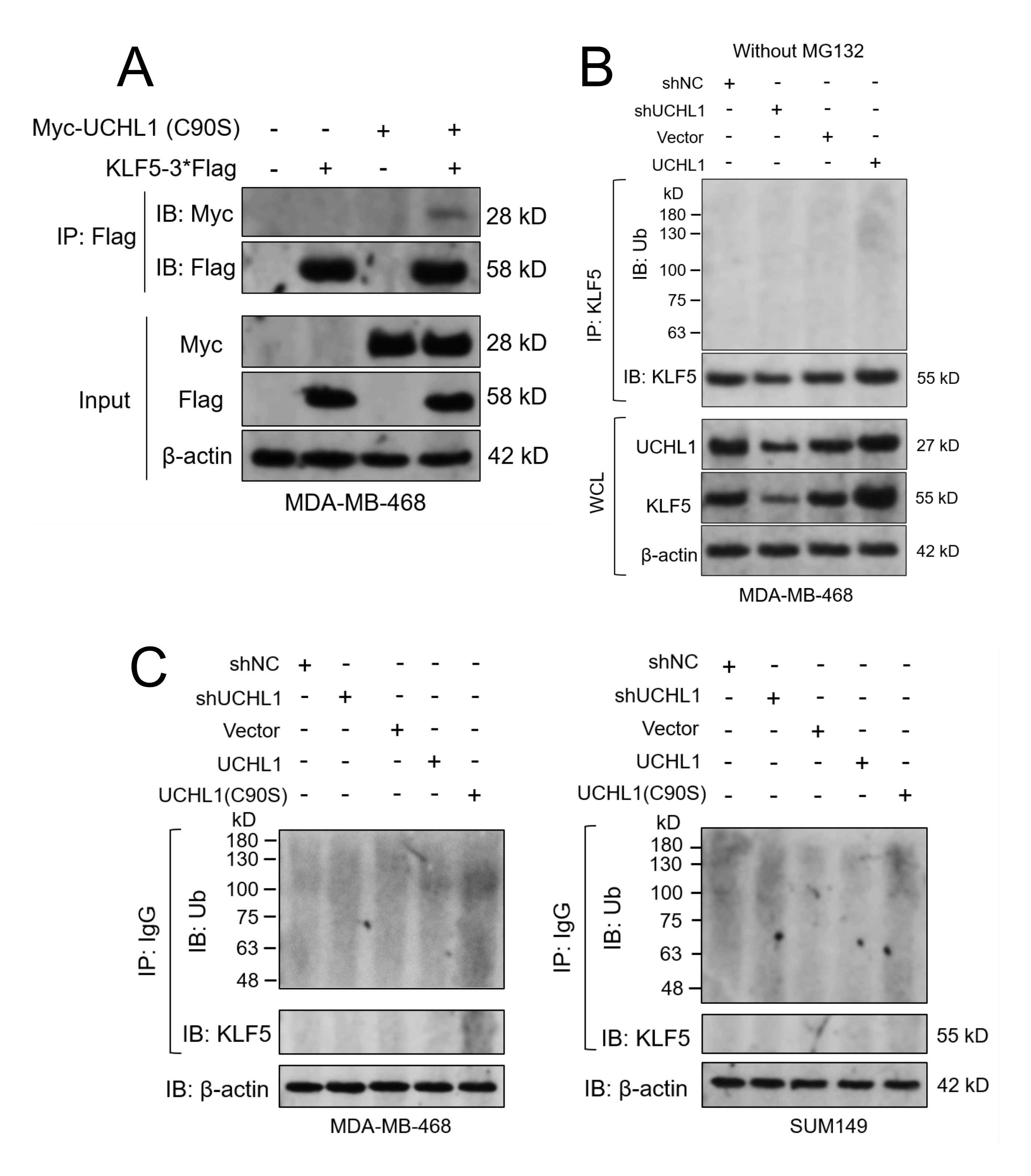

Supplement: Supplementary file 3 — Supplementary Material 3 [file 13058_2024_1800_MOESM3_ESM.tif]

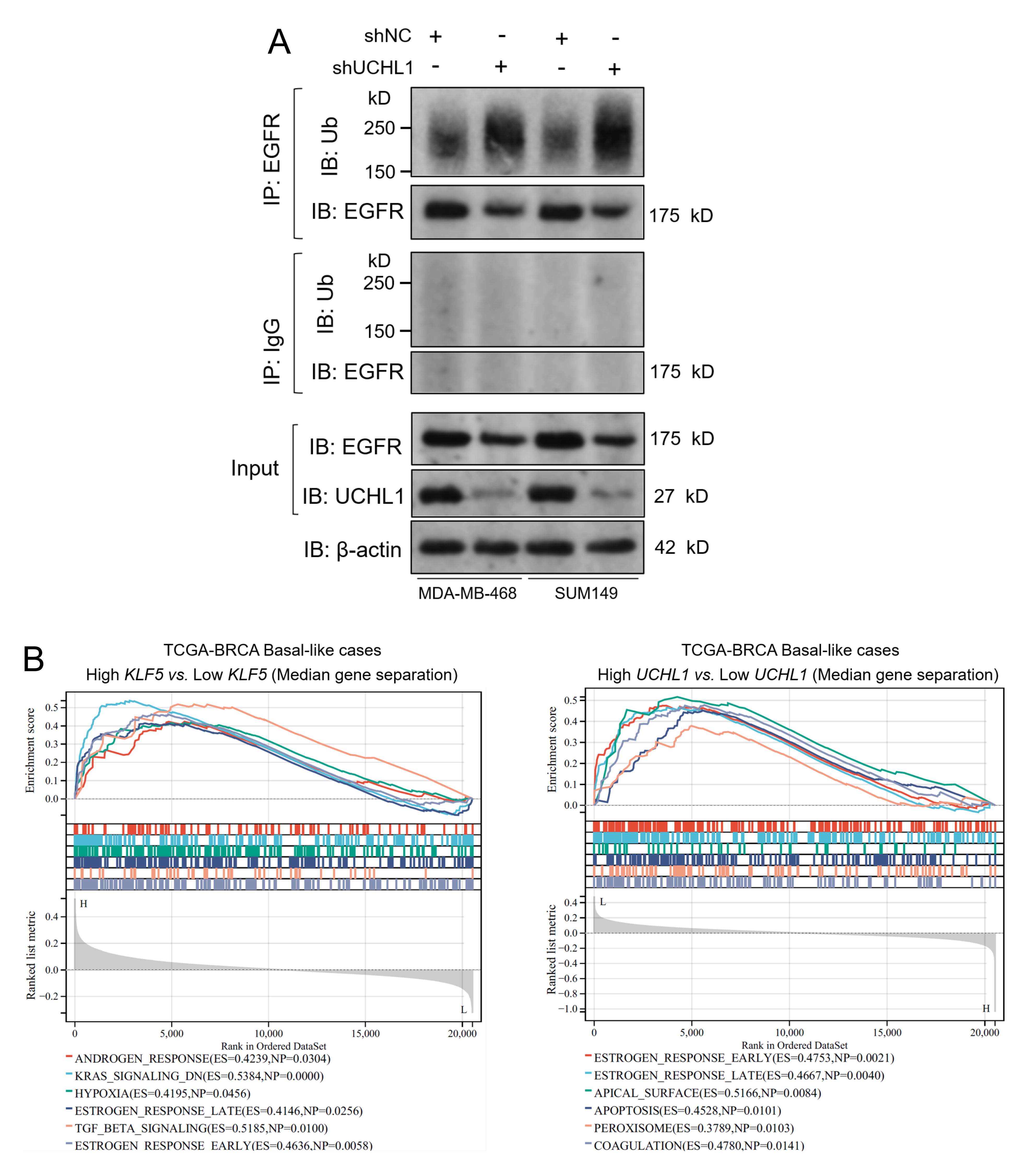

Supplement: Supplementary file 4 — Supplementary Material 4 [file 13058_2024_1800_MOESM4_ESM.tif]

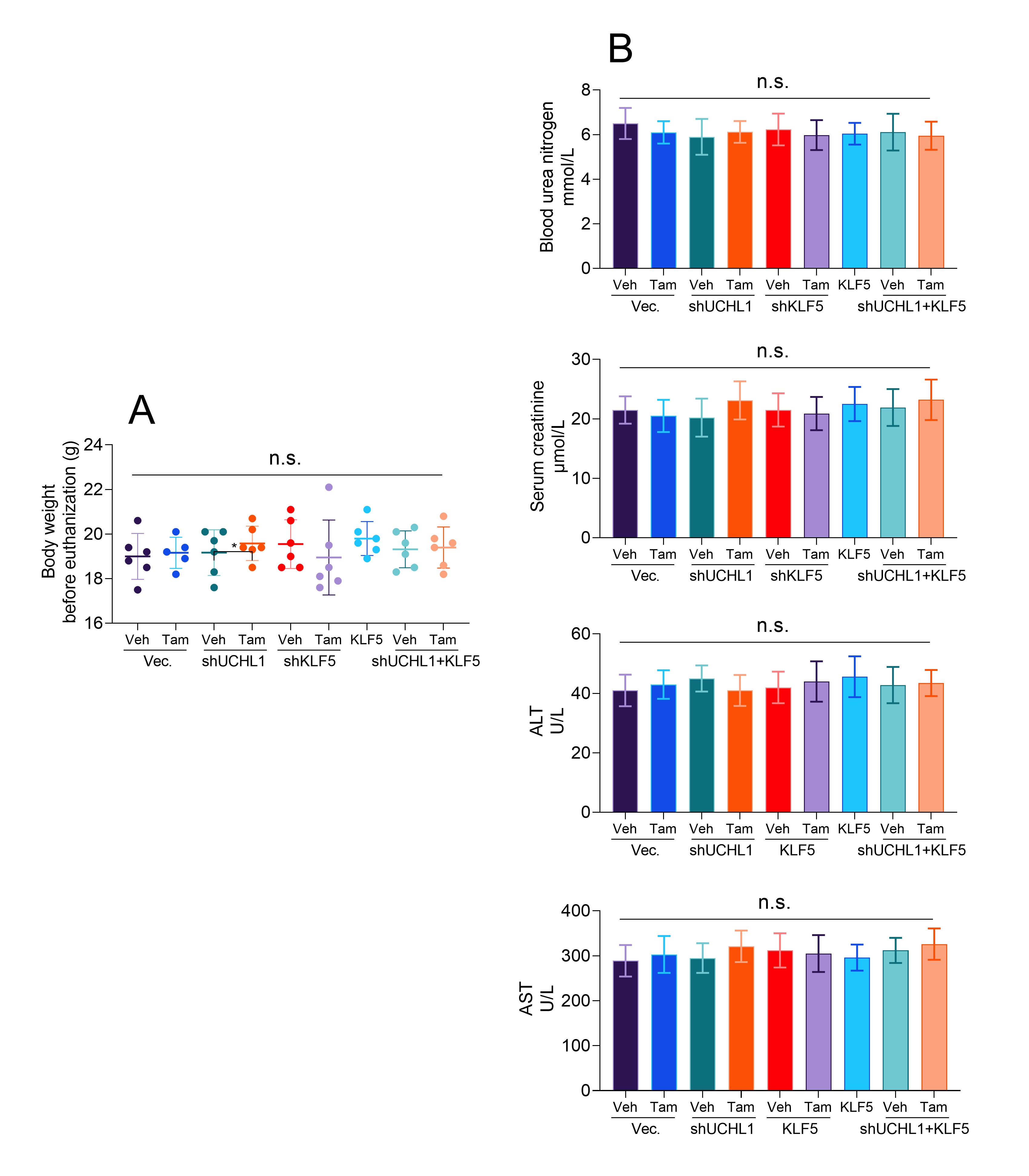

Supplement: Supplementary file 5 — Supplementary Material 5 [file 13058_2024_1800_MOESM5_ESM.tif]

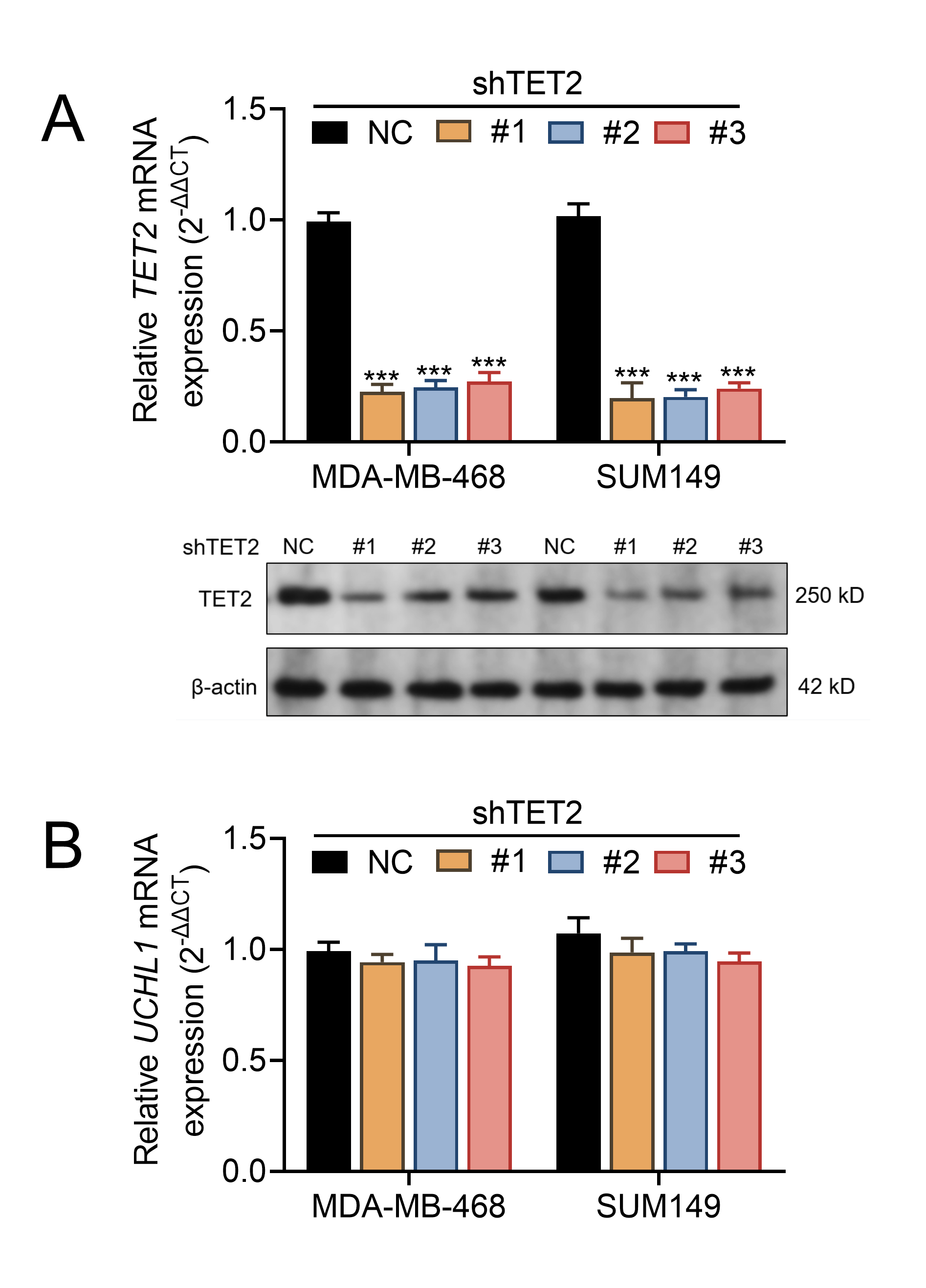

Supplement: Supplementary file 6 — Supplementary Material 6 [file 13058_2024_1800_MOESM6_ESM.tif]

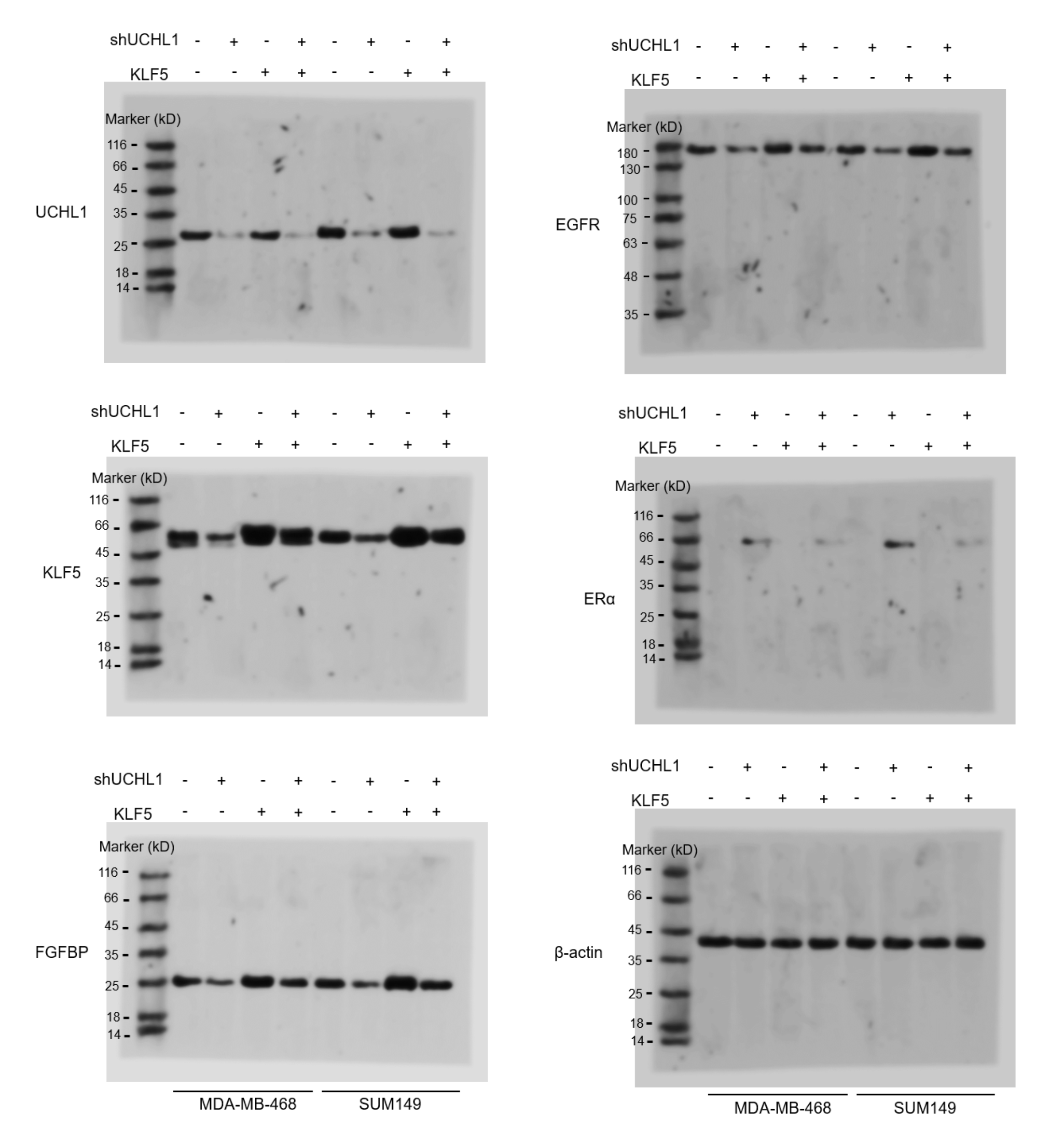

Supplement: Supplementary file 7 — Supplementary Material 7 [file 13058_2024_1800_MOESM7_ESM.tif]

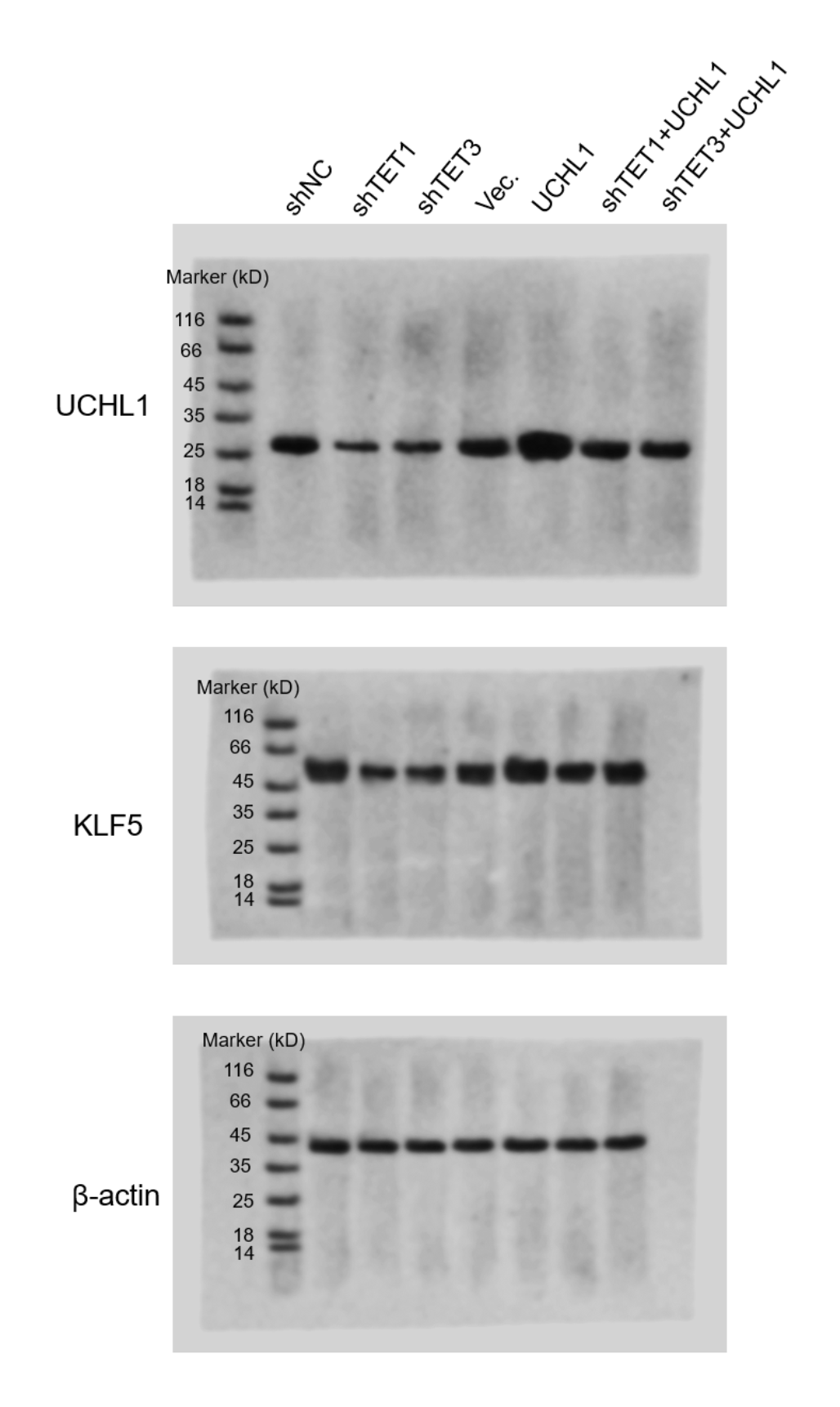

Supplement: Supplementary file 8 — Supplementary Material 8 [file 13058_2024_1800_MOESM8_ESM.tif]

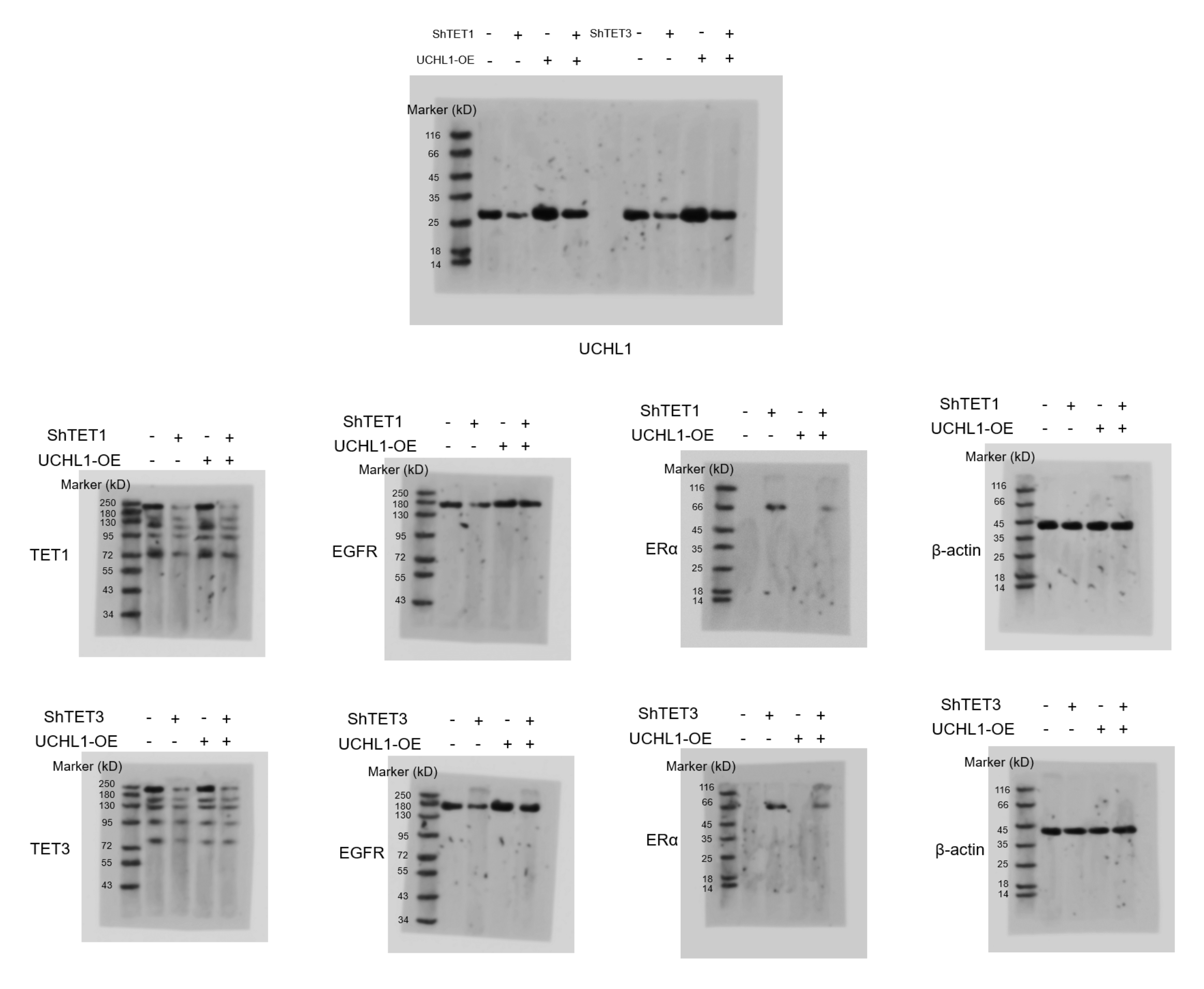

Supplement: Supplementary file 9 — Supplementary Material 9 [file 13058_2024_1800_MOESM9_ESM.tif]
