# Supplementary material for: UCHL1 contributes to insensitivity to endocrine therapy in triple-negative breast cancer by deubiquitinating and stabilizing KLF5
Source: Breast Cancer Res. 2024 Mar 11;26:44. doi: 10.1186/s13058-024-01800-1 (PMC10929172; doi:10.1186/s13058-024-01800-1)

Figure 1C

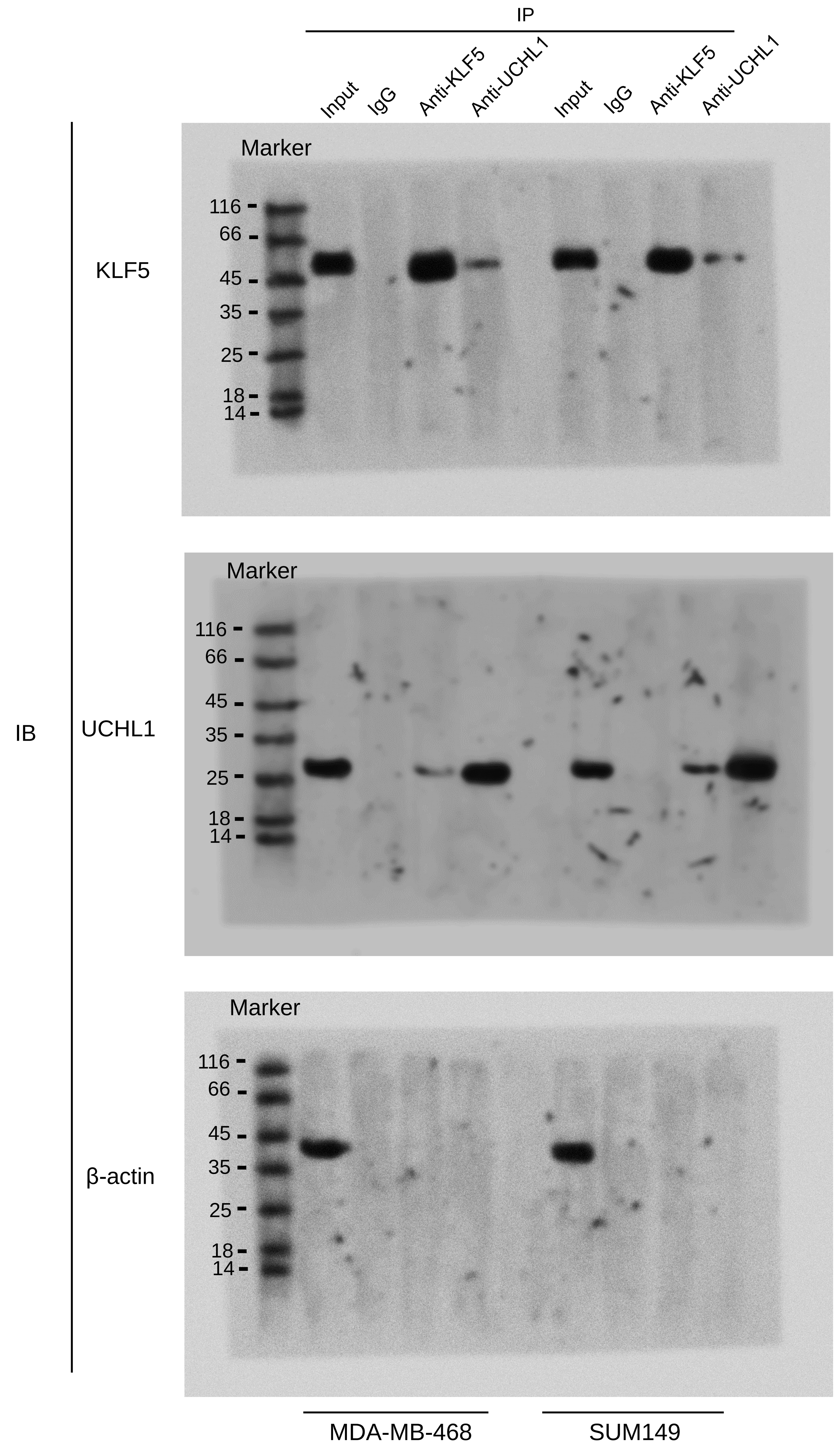

Figure 1D

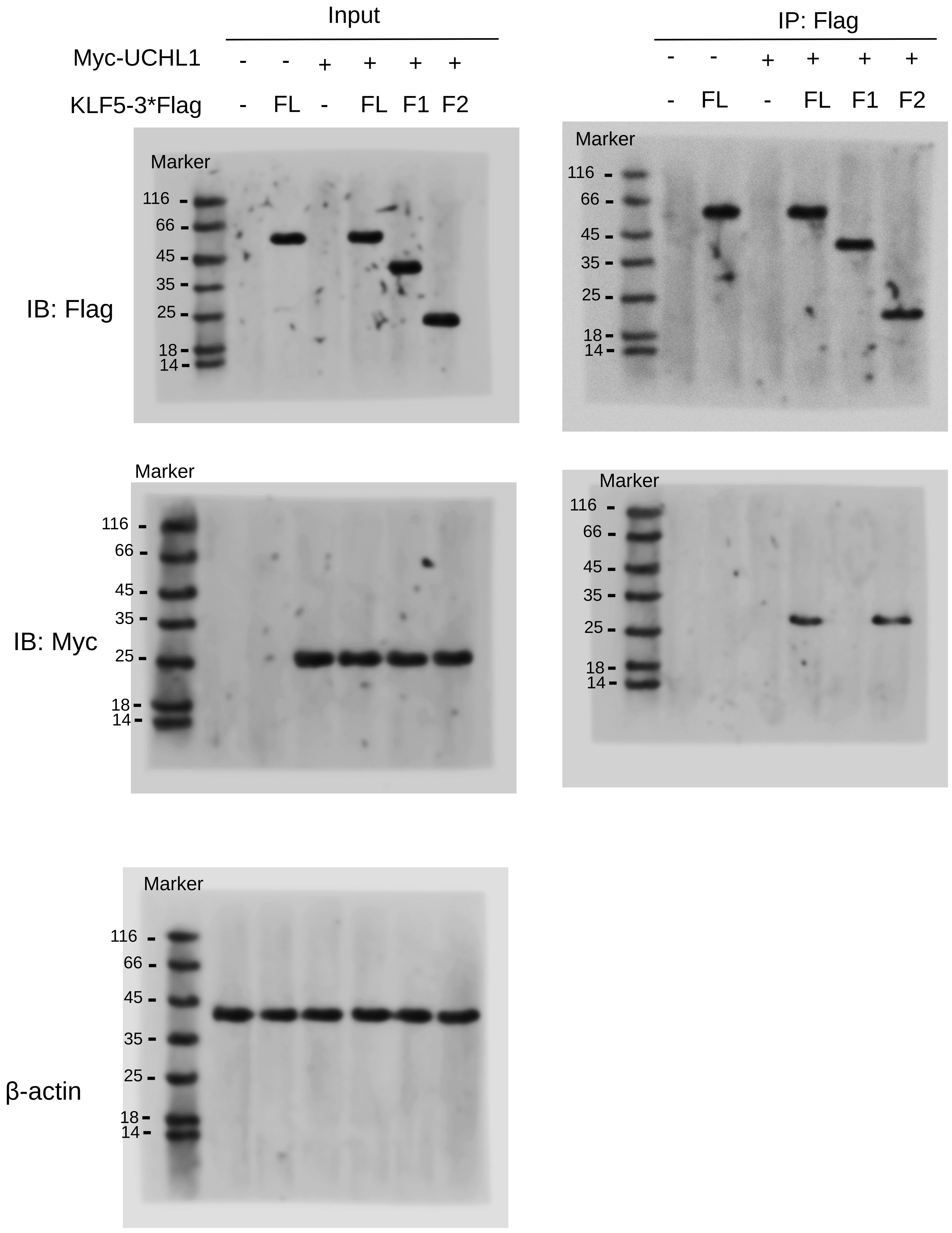

Figure 1E

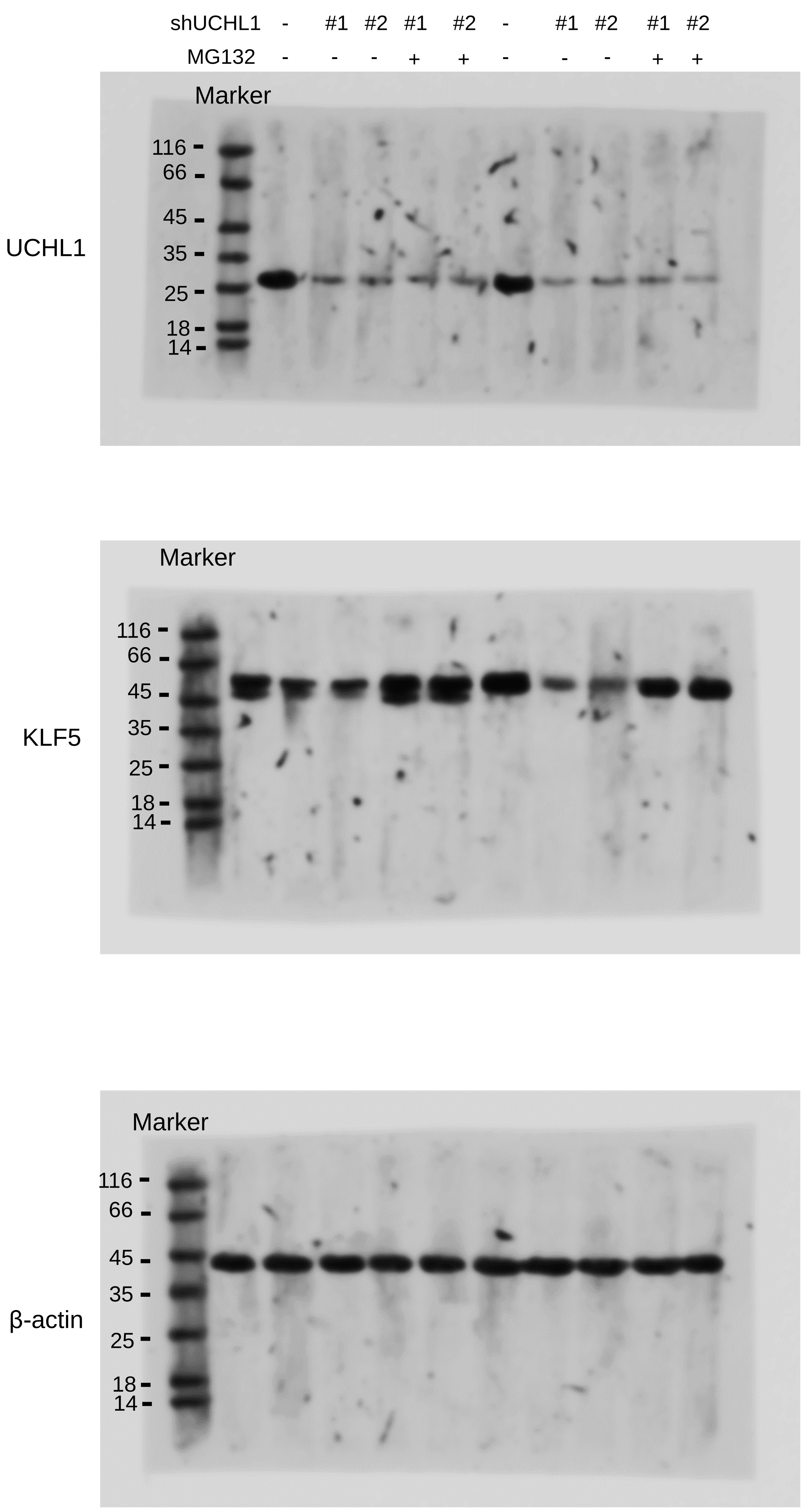

Figure 1F, top two panels

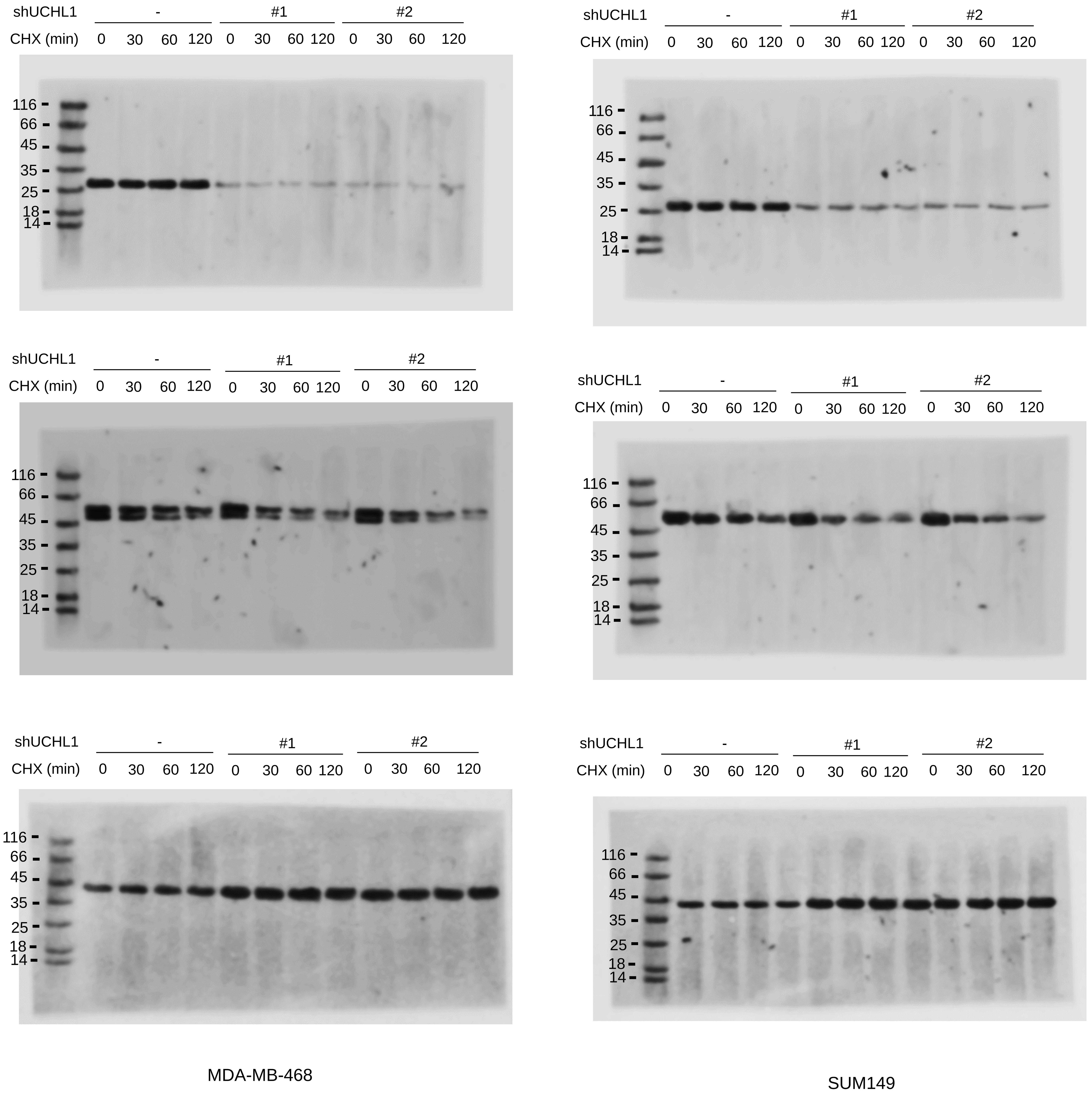

Figure 1F, bottom two panels

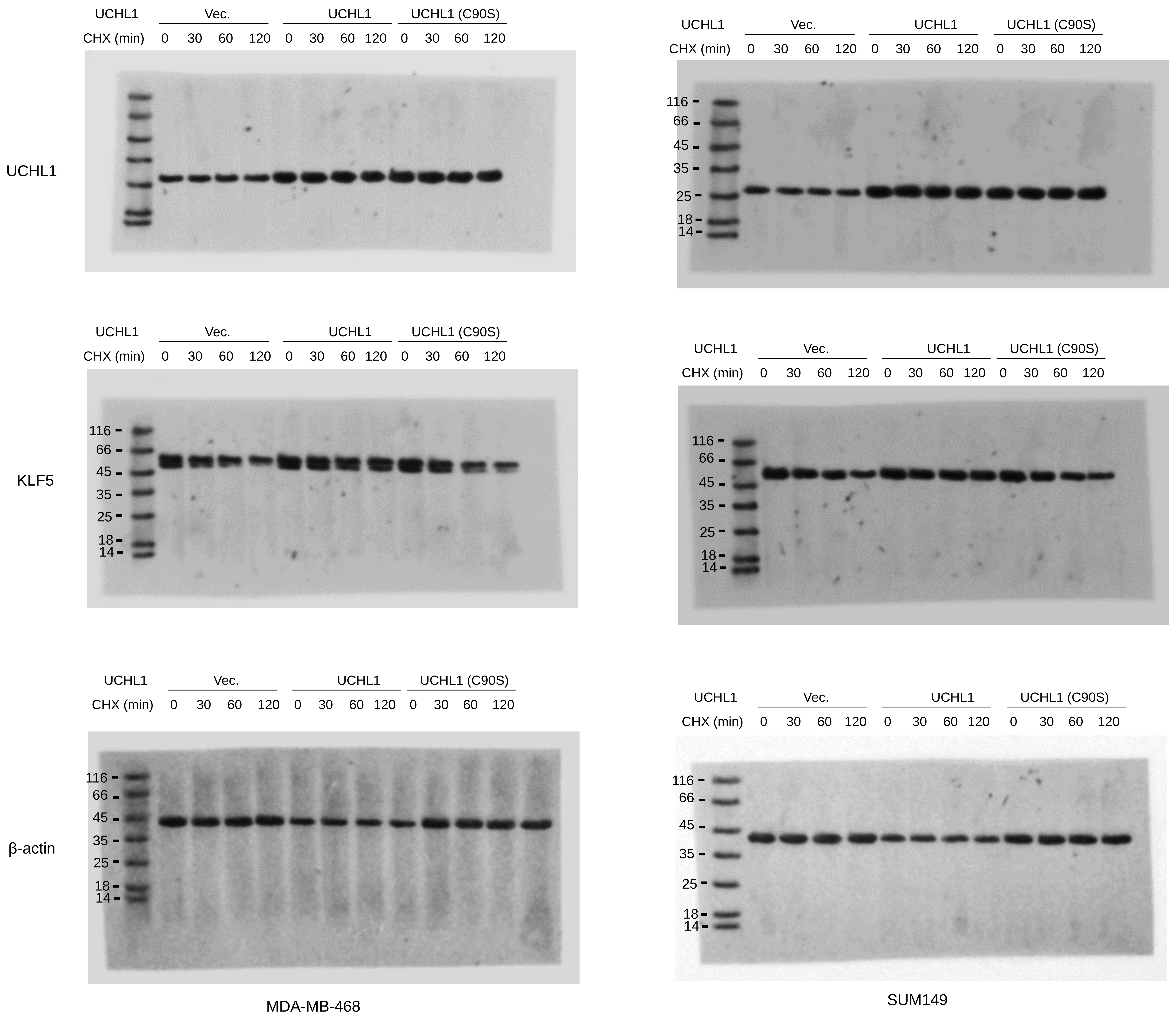

Figure 1H

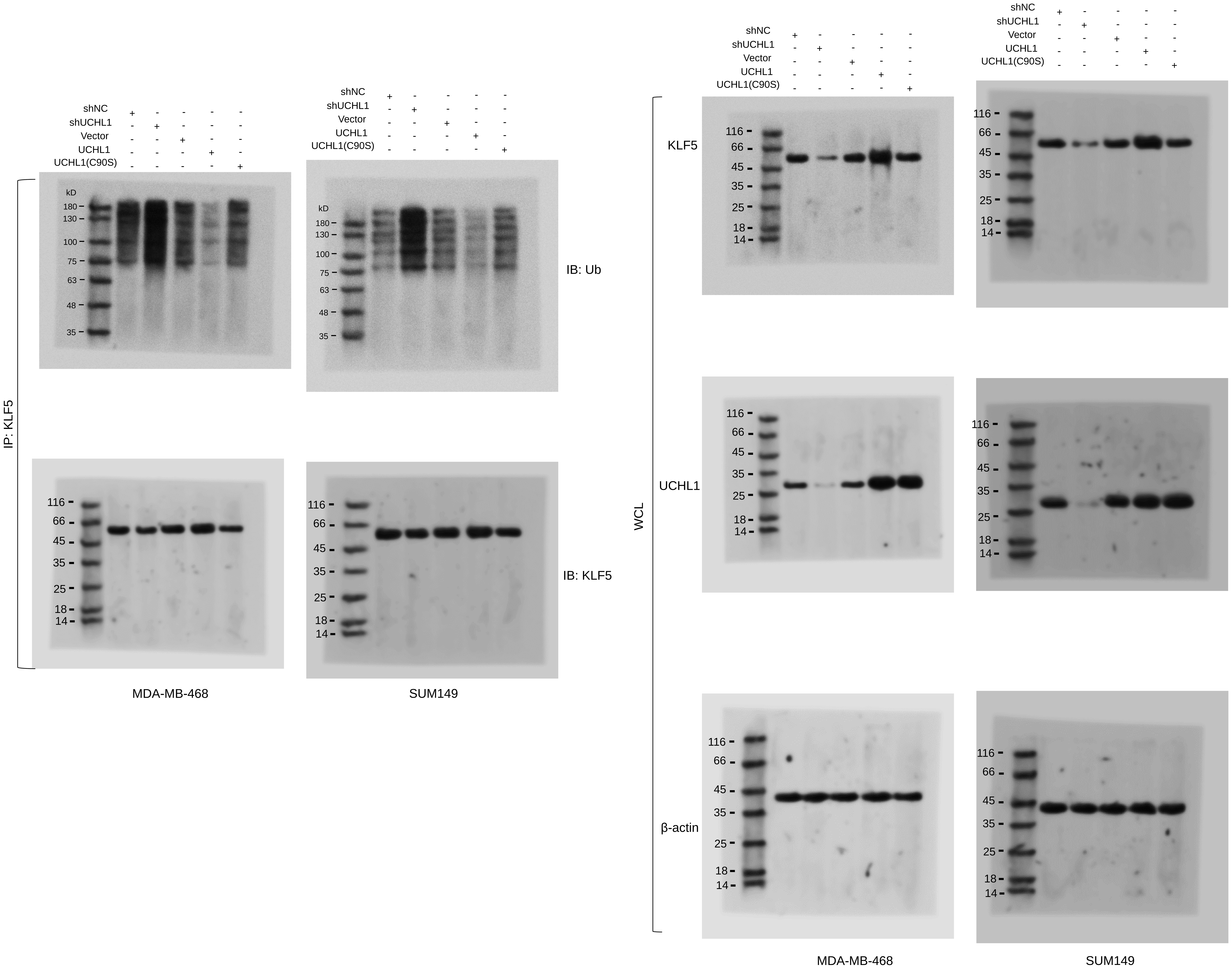
shNC
shUCHL1
Vector
UCHL1
UCHL1(C90S)
IP: KLF5
IB: Ub
IB: KLF5
WCL
KLF5
UCHL1
β-actin
kD
180
130
100
75
63
48
35
116
66
45
25
18
14
MDA-MB-468
SUM149

Figure 2B

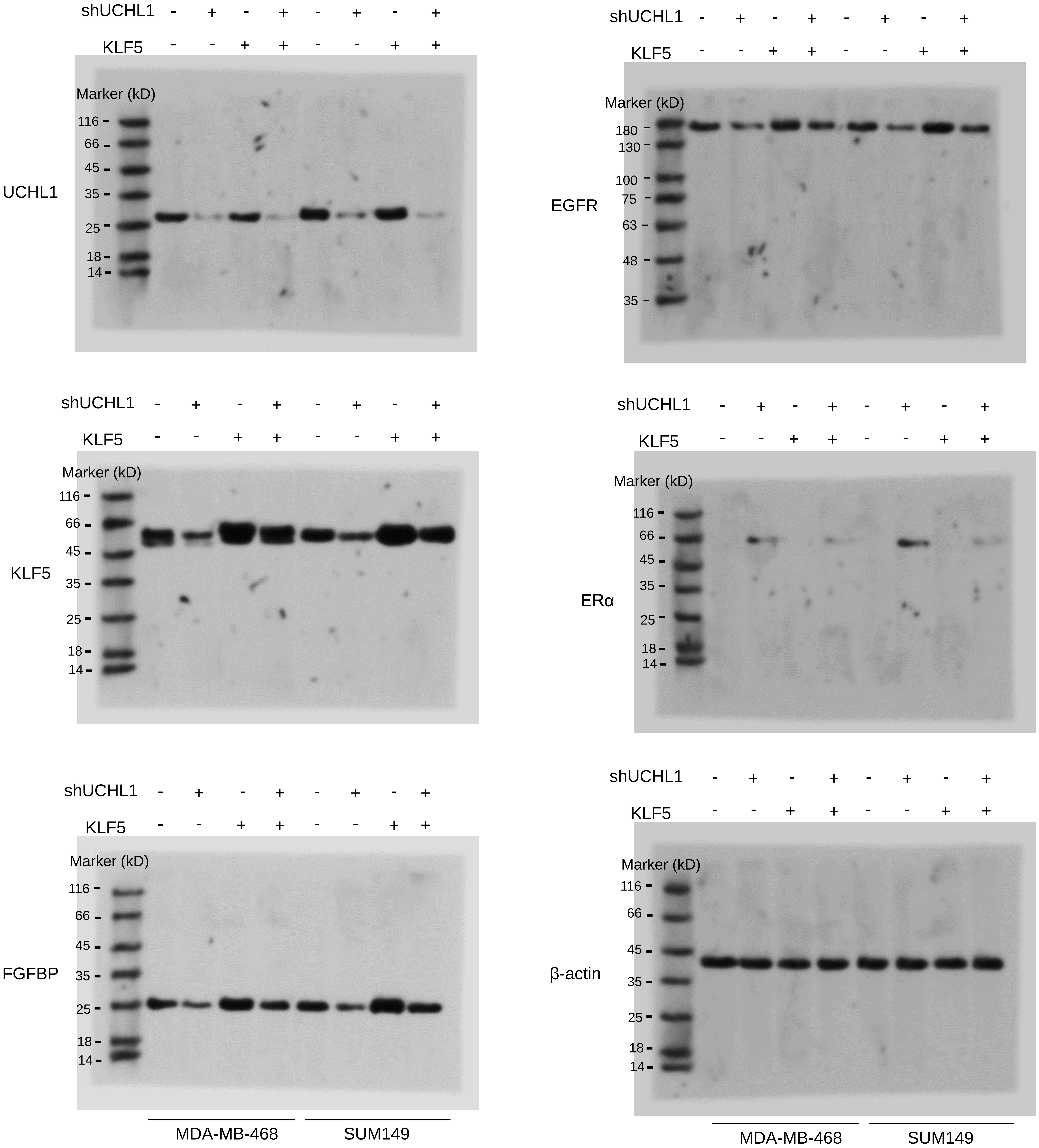
shUCHL1 - + - + - + - +
KLF5 - - + + - - + +
Marker (kD)
116
66
45
35
25
18
14
UCHL1
shUCHL1 - + - + - + - +
KLF5 - - + + - - + +
Marker (kD)
180
130
100
75
63
48
35
EGFR
shUCHL1 - + - + - + - +
KLF5 - - + + - - + +
Marker (kD)
116
66
45
35
25
18
14
KLF5
shUCHL1 - + - + - + - +
KLF5 - - + + - - + +
Marker (kD)
116
66
45
35
25
18
14
ERα
shUCHL1 - + - + - + - +
KLF5 - - + + - - + +
Marker (kD)
116
66
45
35
25
18
14
FGFBP
MDA-MB-468
SUM149
shUCHL1 - + - + - + - +
KLF5 - - + + - - + +
Marker (kD)
116
66
45
35
25
18
14
β-actin
MDA-MB-468
SUM149

Figure 4H

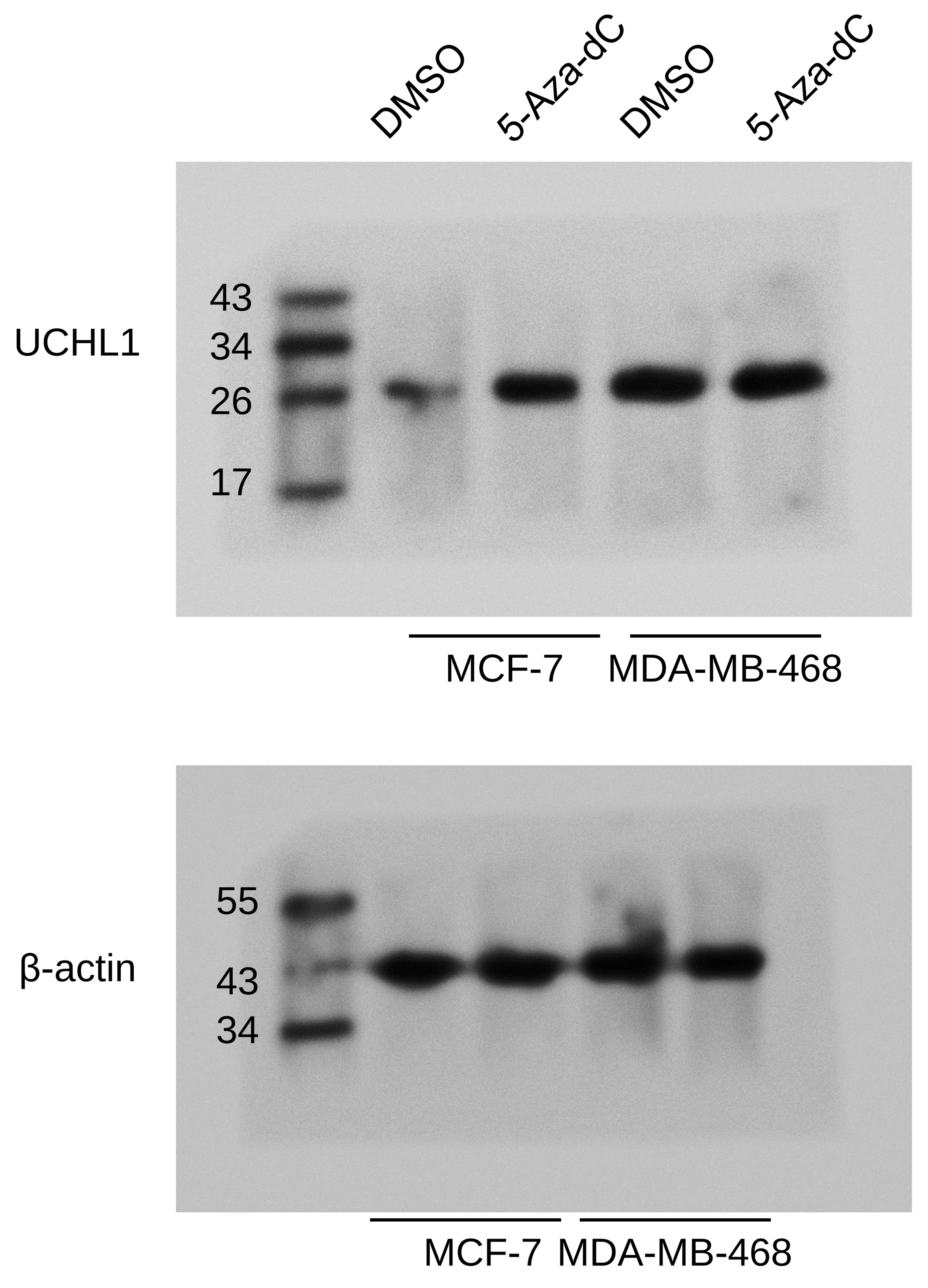

Figure 6D

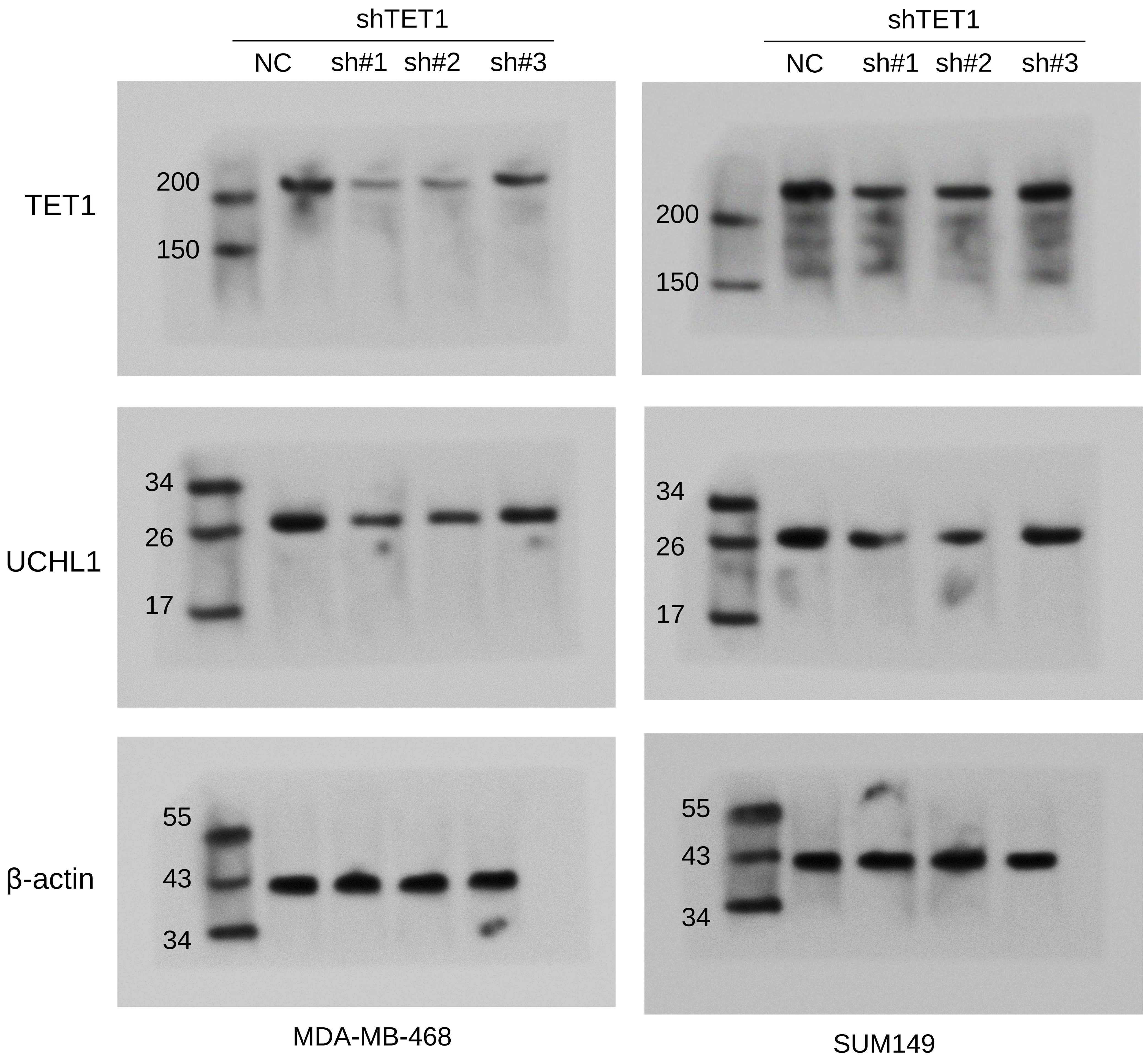

Figure 6E

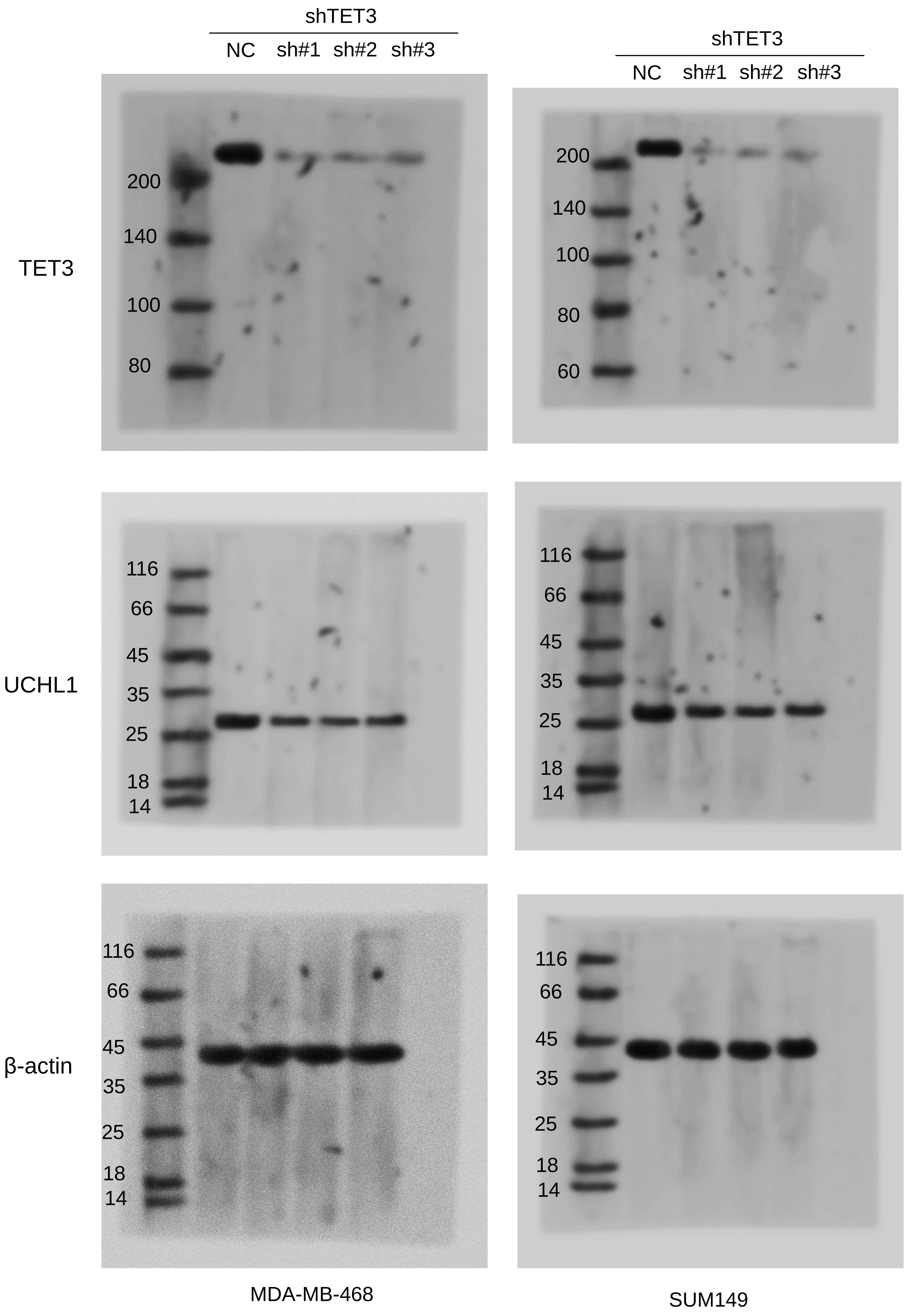

Figure 6I

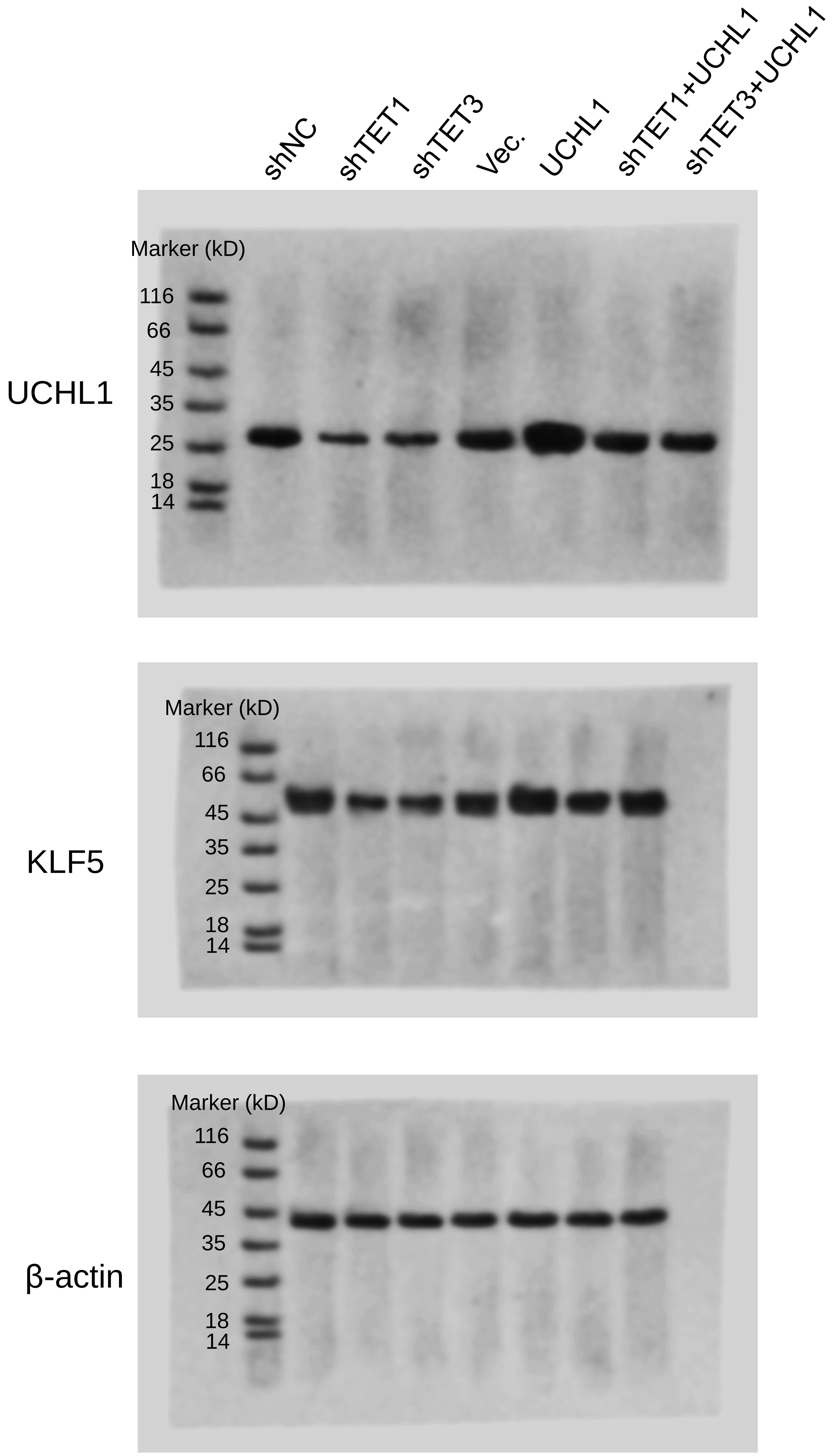
shNC
shTET1
shTET3
Vec.
UCHL1
shTET1+UCHL1
shTET3+UCHL1
UCHL1
Marker (kD)
116
66
45
35
25
18
14
KLF5
Marker (kD)
116
66
45
35
25
18
14
β-actin
Marker (kD)
116
66
45
35
25
18
14

Figure 6K

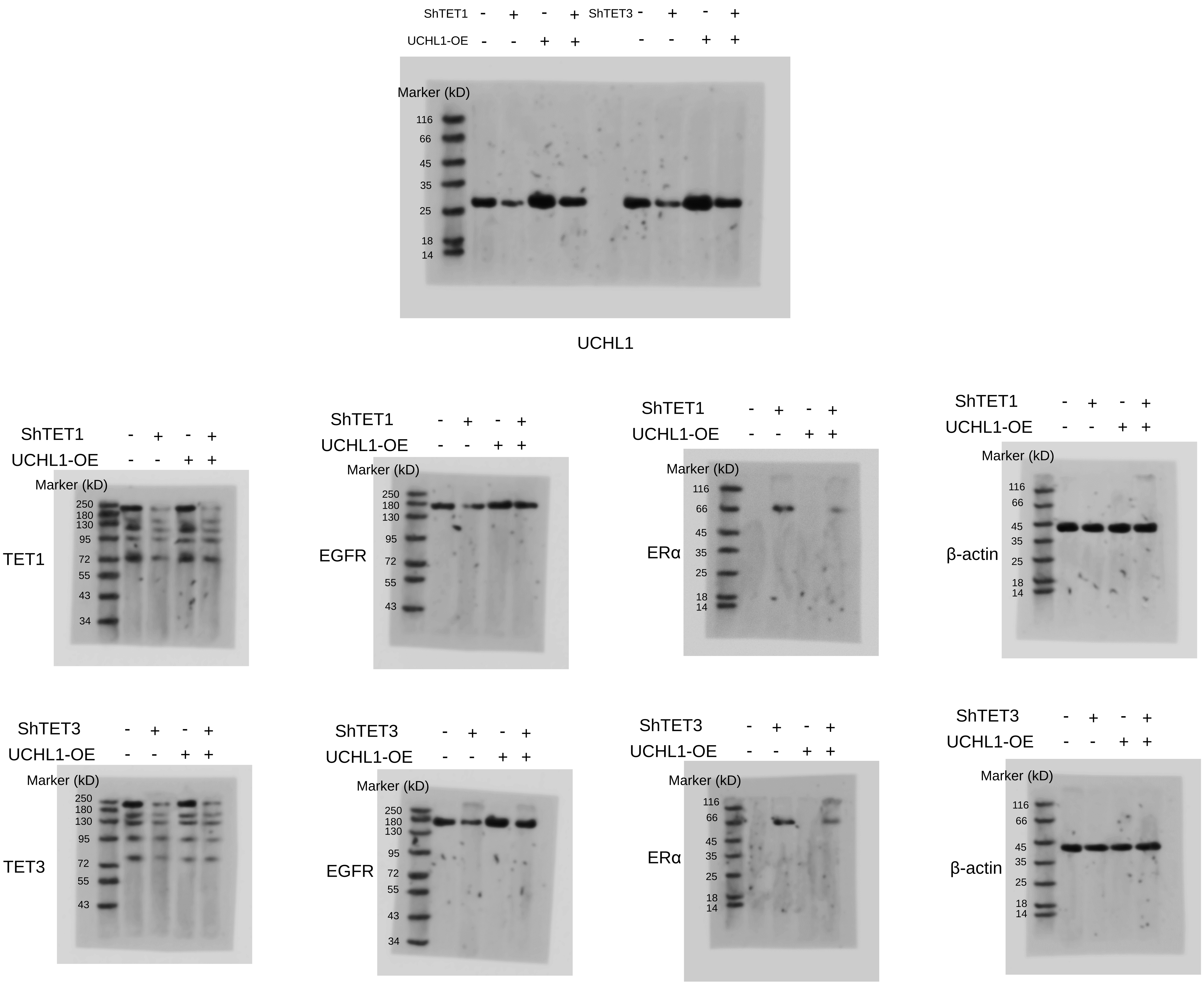
ShTET1 - + - + ShTET3 - + - +
UCHL1-OE - - + + - - + +
Marker (kD)
116
66
45
35
25
18
14
UCHL1
ShTET1 - + - +
UCHL1-OE - - + +
Marker (kD)
250
180
130
95
72
55
43
34
TET1
EGFR
ERα
β-actin
ShTET3 - + - +
UCHL1-OE - - + +
TET3
EGFR
ERα
β-actin

Supplementary Figure 2B

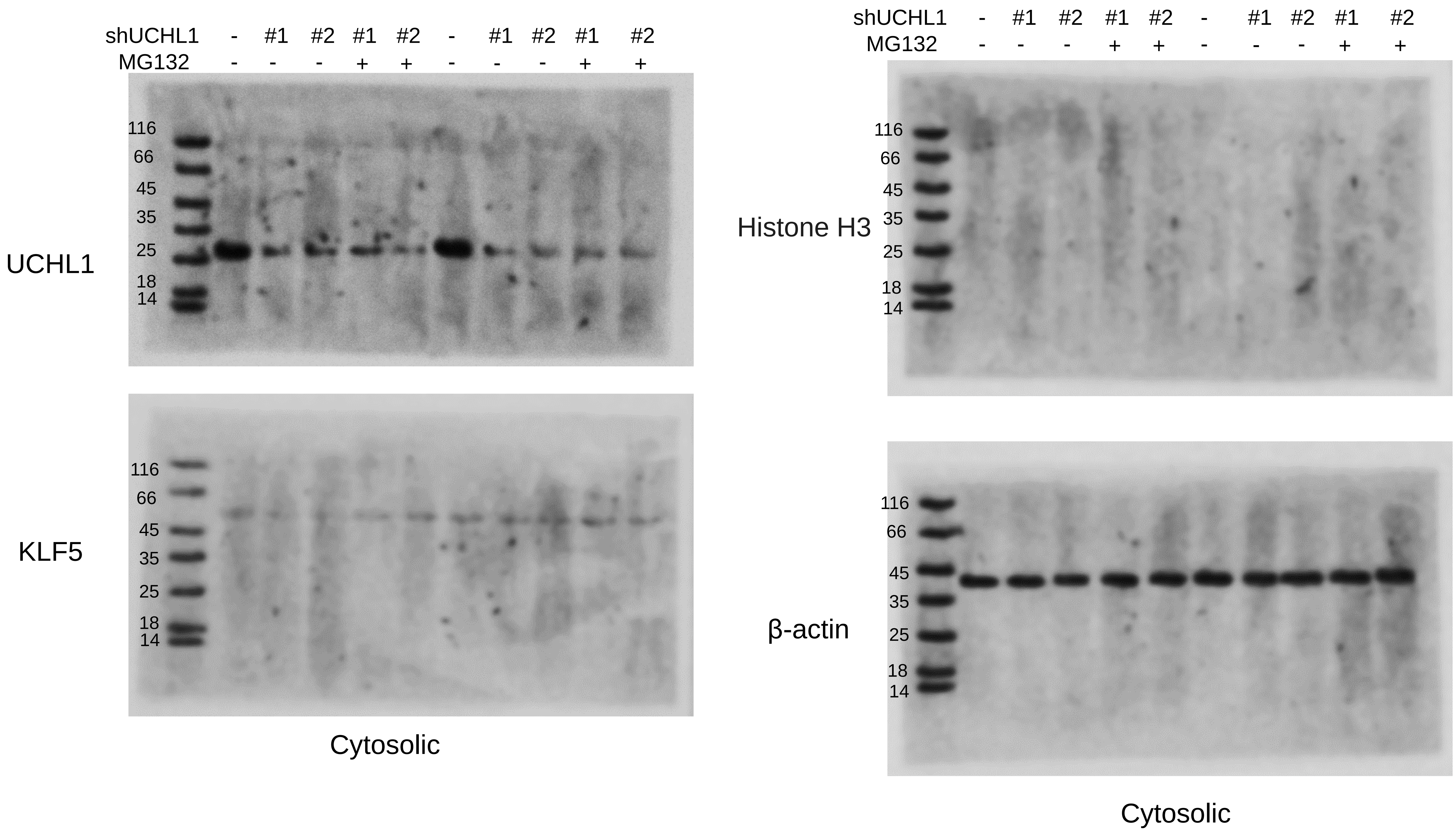

Supplementary Figure 2B

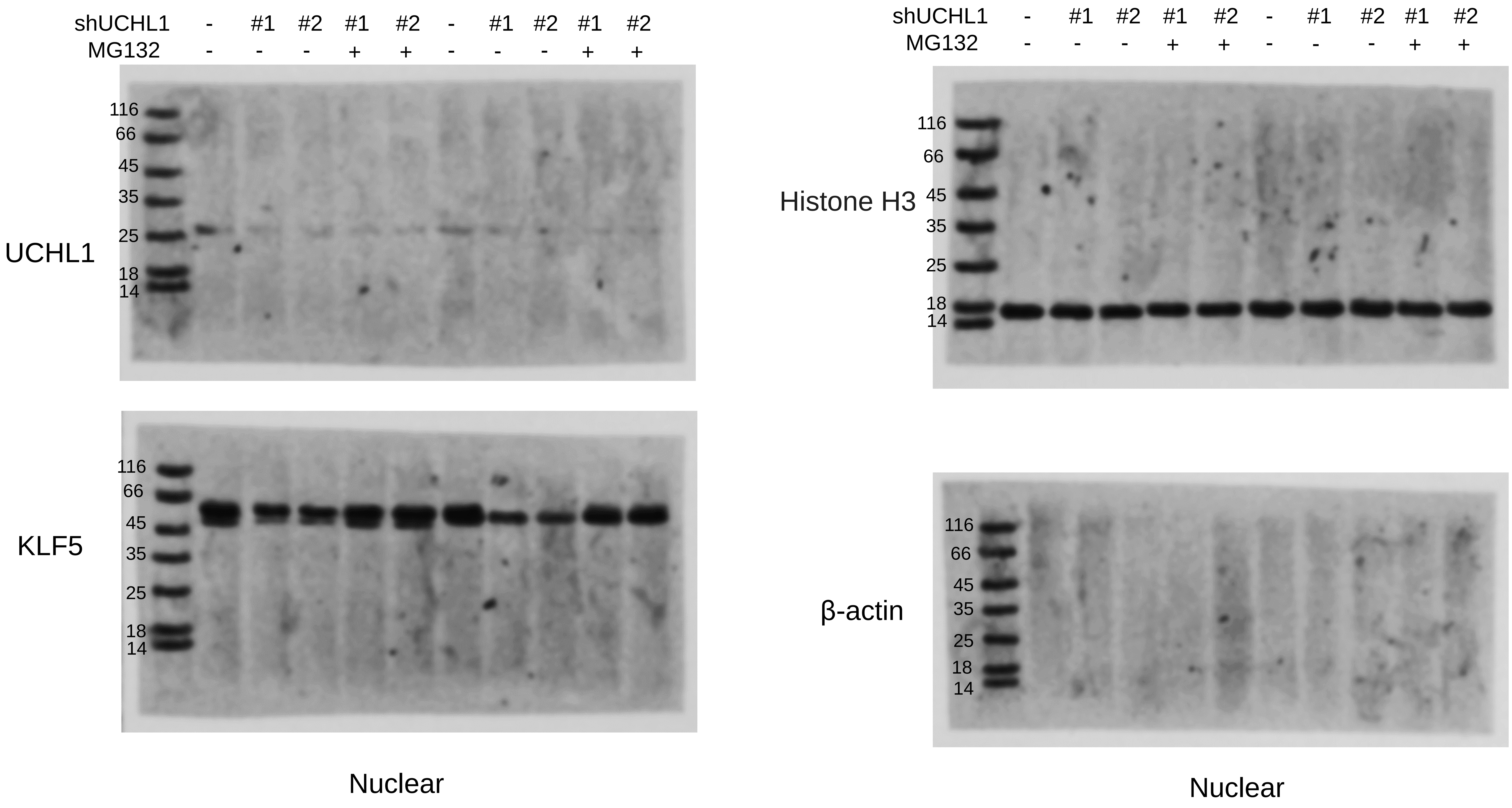

Supplementary Figure 3A

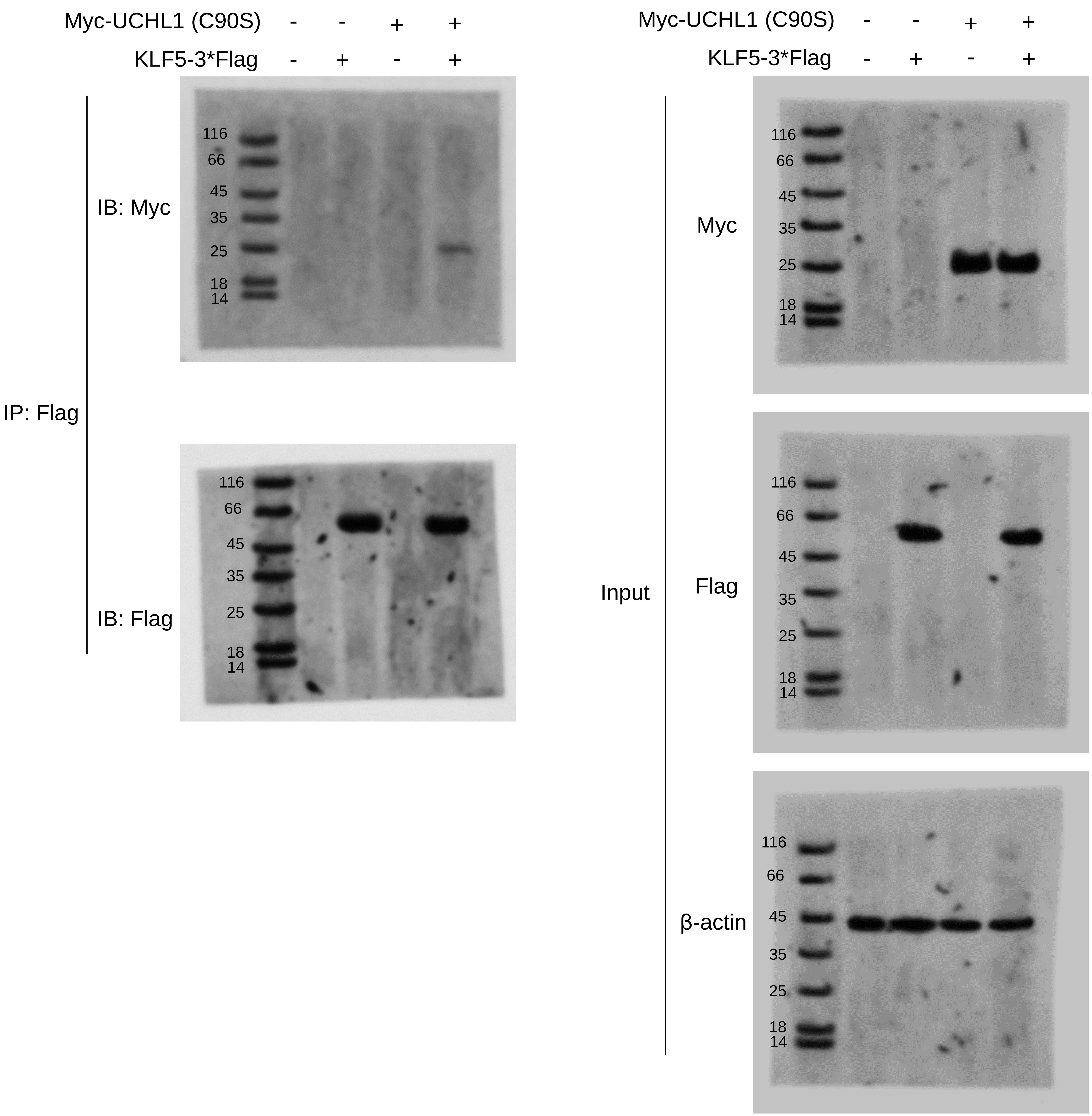

Supplementary Figure 3B

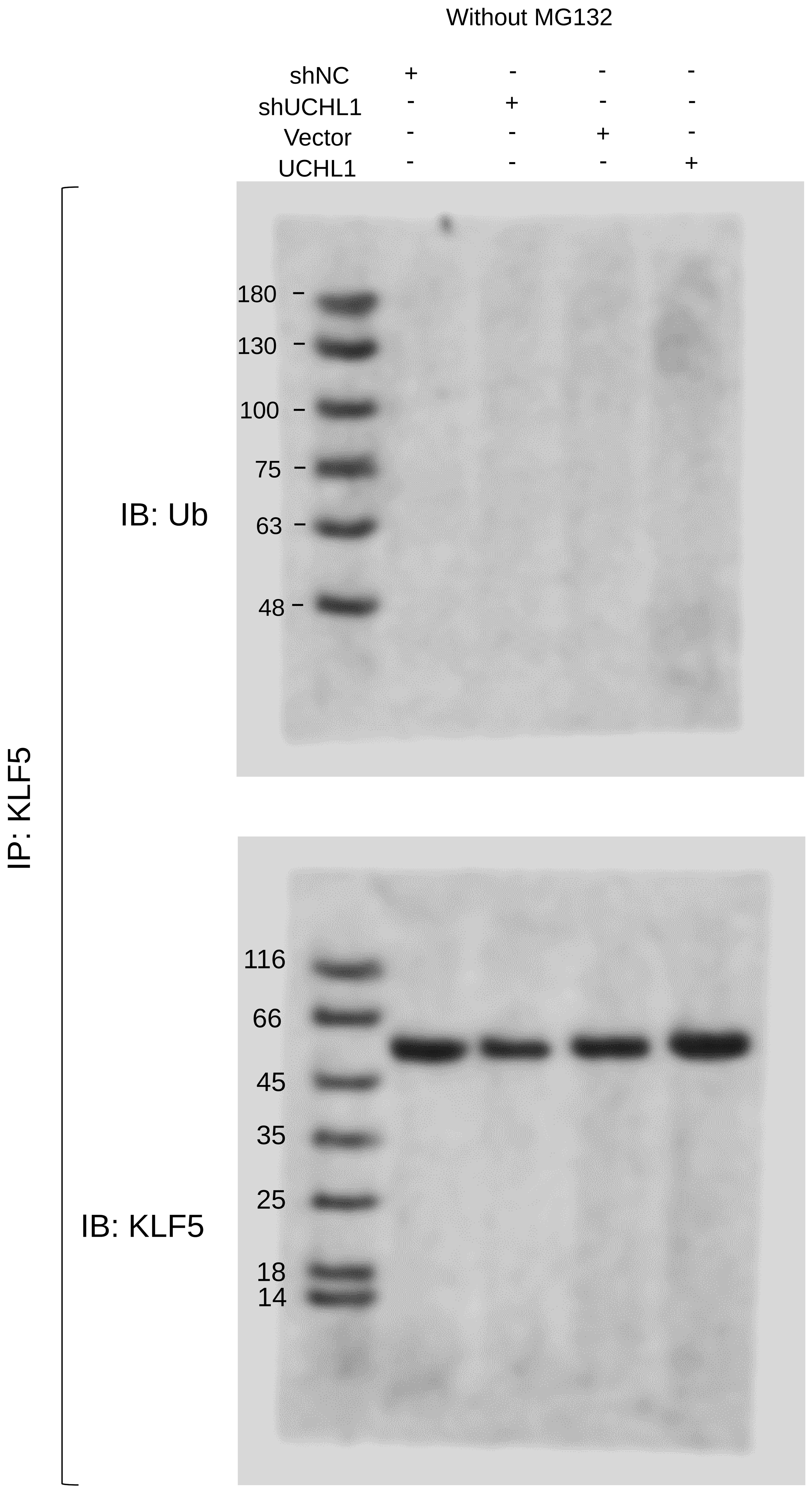

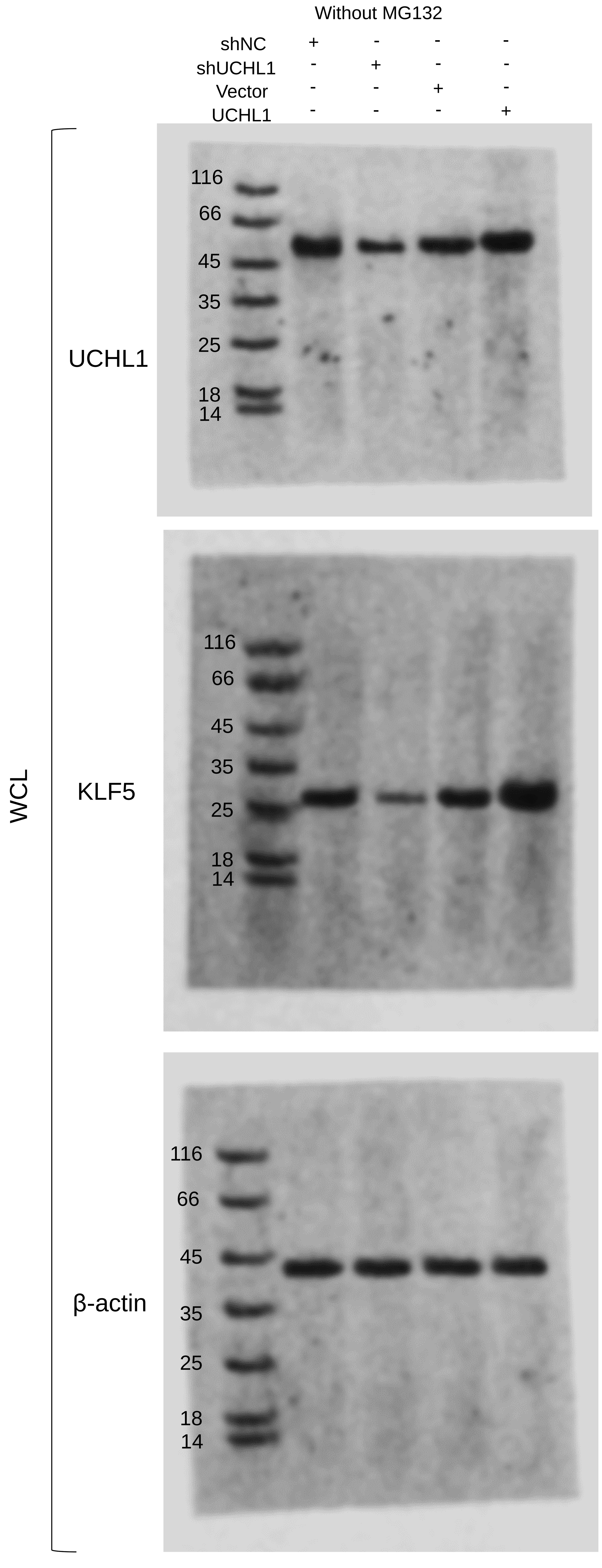

Supplementary Figure 3C

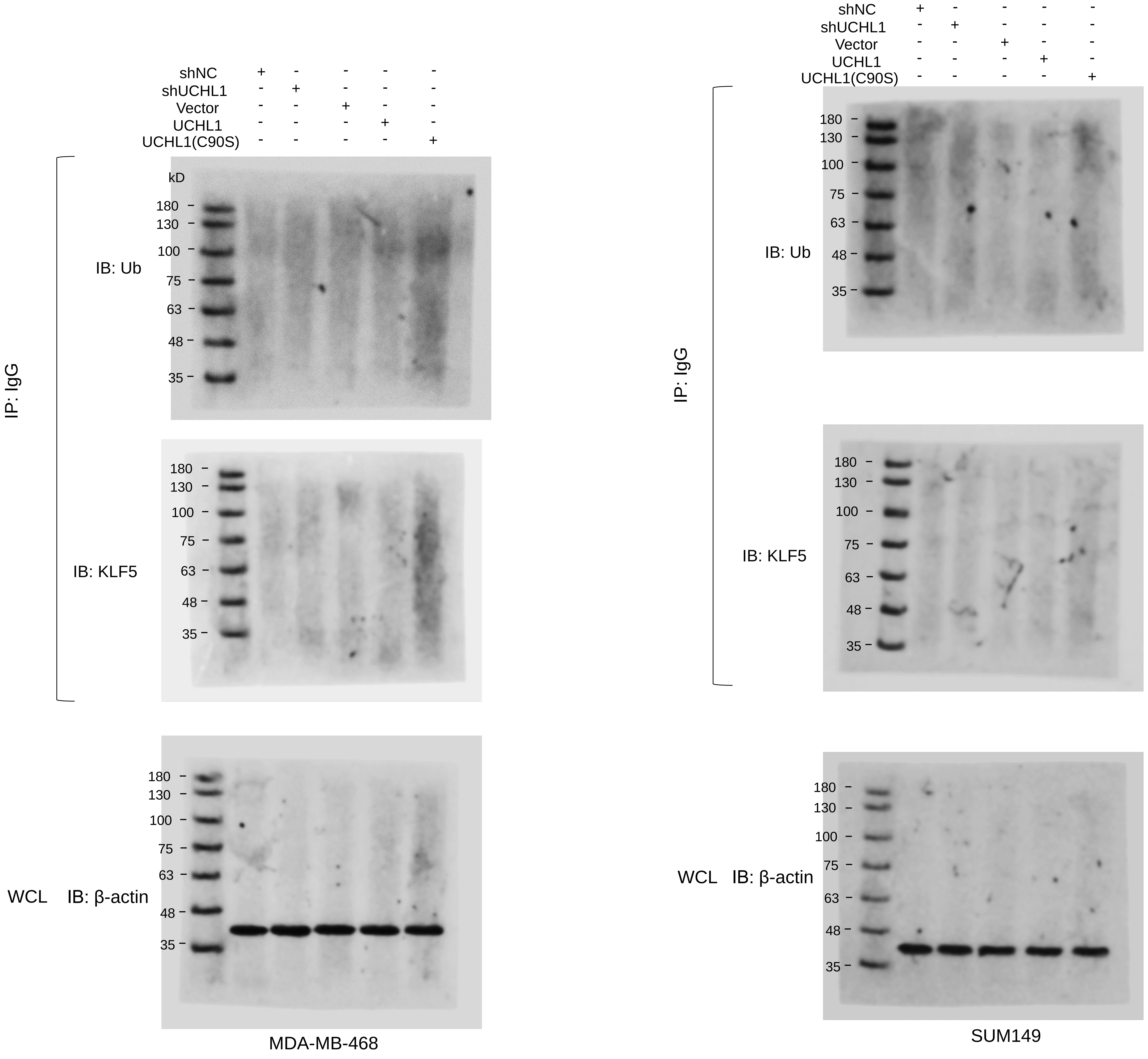

Supplementary Figure 4A

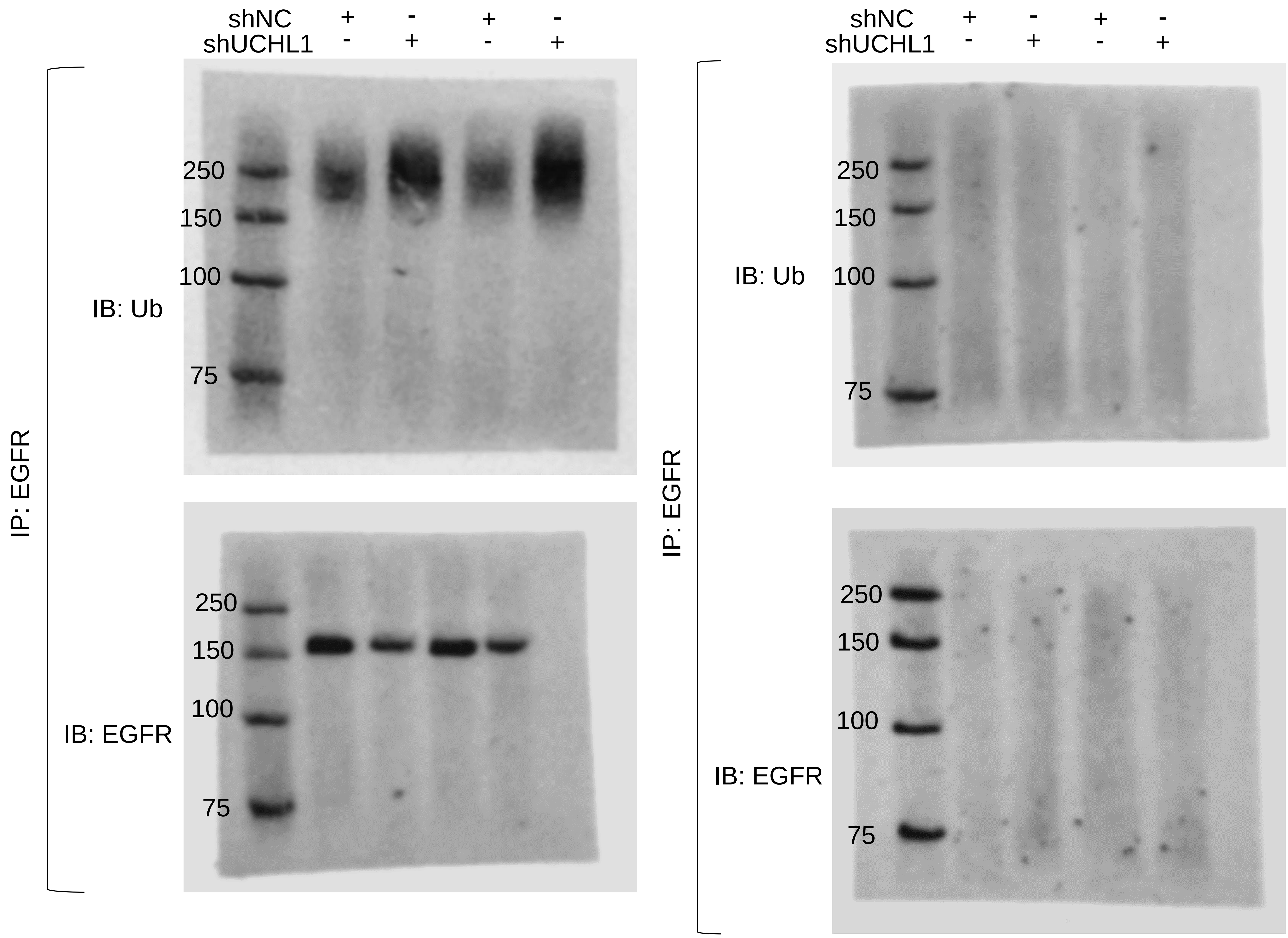

Supplementary Figure 4A

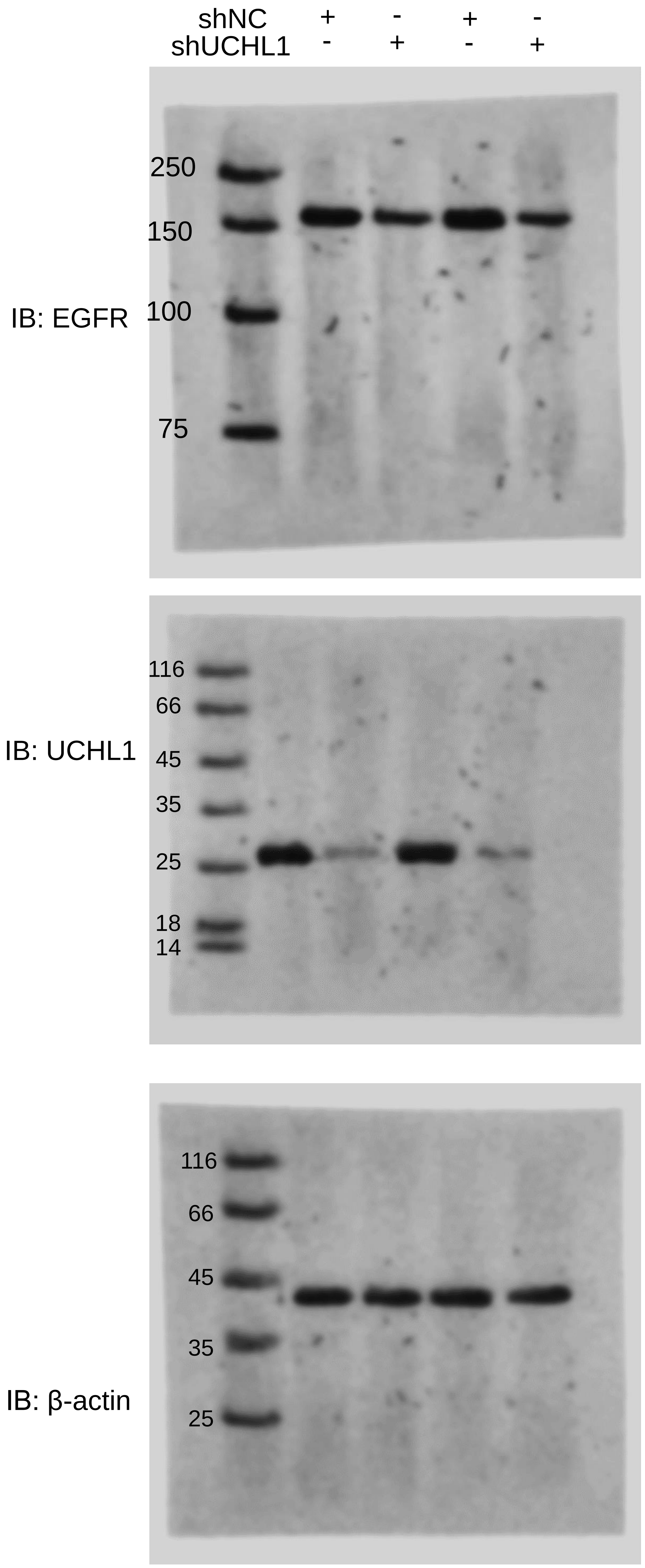

Supplementary Figure 6

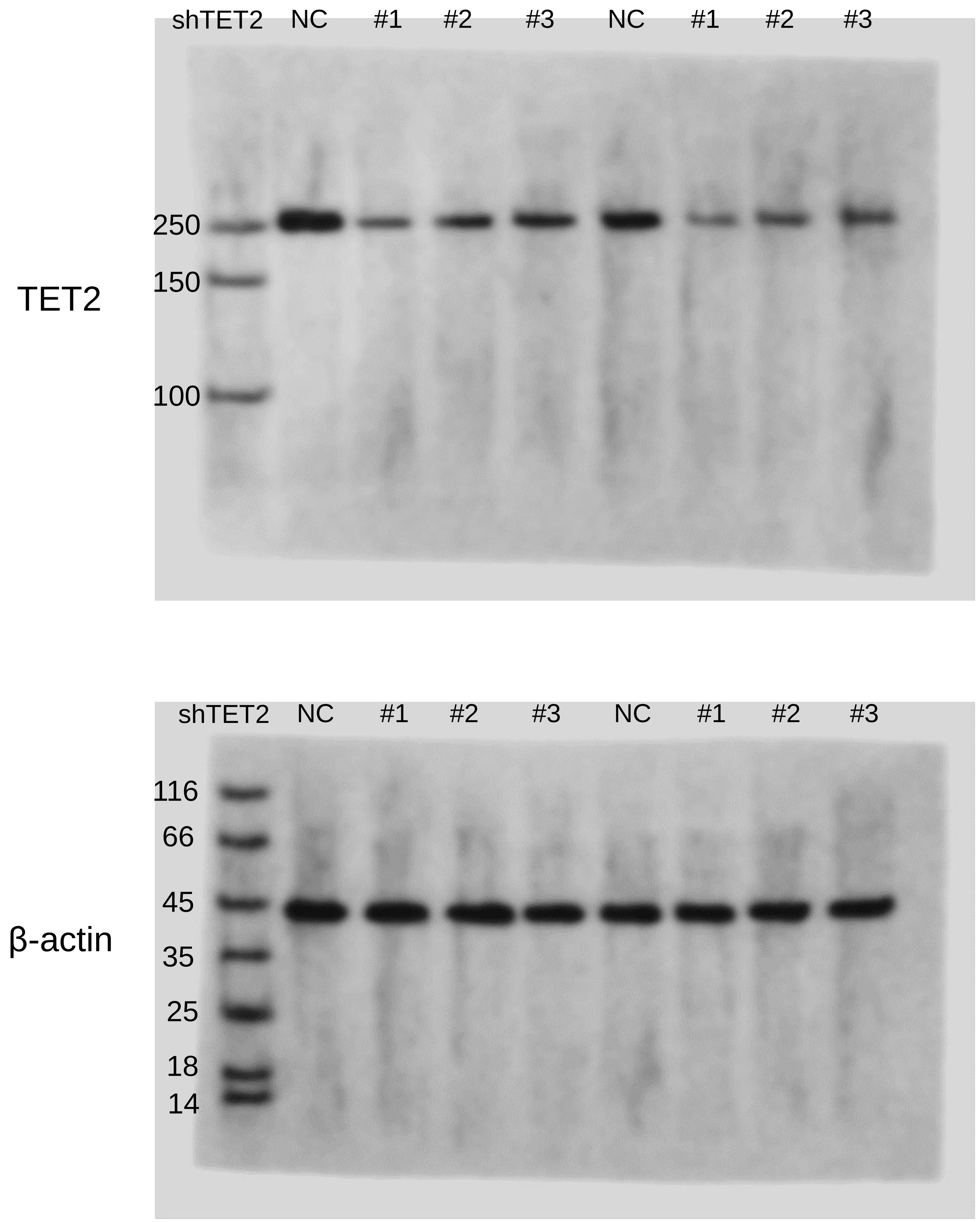

Supplement: Supplementary file 10 — Supplementary Material 10 [file 13058_2024_1800_MOESM10_ESM.pdf]
